# Protein lysine acetylation plays a regulatory role in *Bacillus subtilis* multicellularity

**DOI:** 10.1371/journal.pone.0204687

**Published:** 2018-09-28

**Authors:** Alicyn Reverdy, Yun Chen, Evan Hunter, Kevin Gozzi, Yunrong Chai

**Affiliations:** 1 Department of Biology, Northeastern University, Boston, MA, United States of America; 2 Institute of Biotechnology, Zhejiang University, Hangzhou, China; University of Florida, UNITED STATES

## Abstract

Protein lysine acetylation is a post-translational modification that alters the charge, conformation, and stability of proteins. A number of genome-wide characterizations of lysine-acetylated proteins, or acetylomes, in bacteria have demonstrated that lysine acetylation occurs on proteins with a wide diversity of functions, including central metabolism, transcription, chemotaxis, and cell size regulation. *Bacillus subtilis* is a model organism for studies of sporulation, motility, cell signaling, and multicellular development (or biofilm formation). In this work, we investigated the role of global protein lysine acetylation in multicellular development in *B*. *subtilis*. We analyzed the *B*. *subtilis* acetylome under biofilm-inducing conditions and identified acetylated proteins involved in multicellularity, specifically, swarming and biofilm formation. We constructed various single and double mutants of genes known to encode enzymes involved in global protein lysine acetylation in *B*. *subtilis*. Some of those mutants showed a defect in swarming motility while others demonstrated altered biofilm phenotypes. Lastly, we picked two acetylated proteins known to be important for biofilm formation, YmcA (also known as RicA), a regulatory protein critical for biofilm induction, and GtaB, an UTP-glucose-1-phosphate uridylyltransferase that synthesizes a nucleotide sugar precursor for biosynthesis of exopolysaccharide, a key biofilm matrix component. We performed site-directed mutagenesis on the acetylated lysine codons in *ymcA* and *gtaB*, respectively, and assayed cells bearing those point mutants for biofilm formation. The mutant alleles of *ymcA*(K64R), *gtaB*(K89R), and *gtaB*(K191R) all demonstrated a severe biofilm defect. These results indicate the importance of acetylated lysine residues in both YmcA and GtaB. In summary, we propose that protein lysine acetylation plays a global regulatory role in *B*. *subtilis* multicellularity.

## Introduction

**Fig 1:**
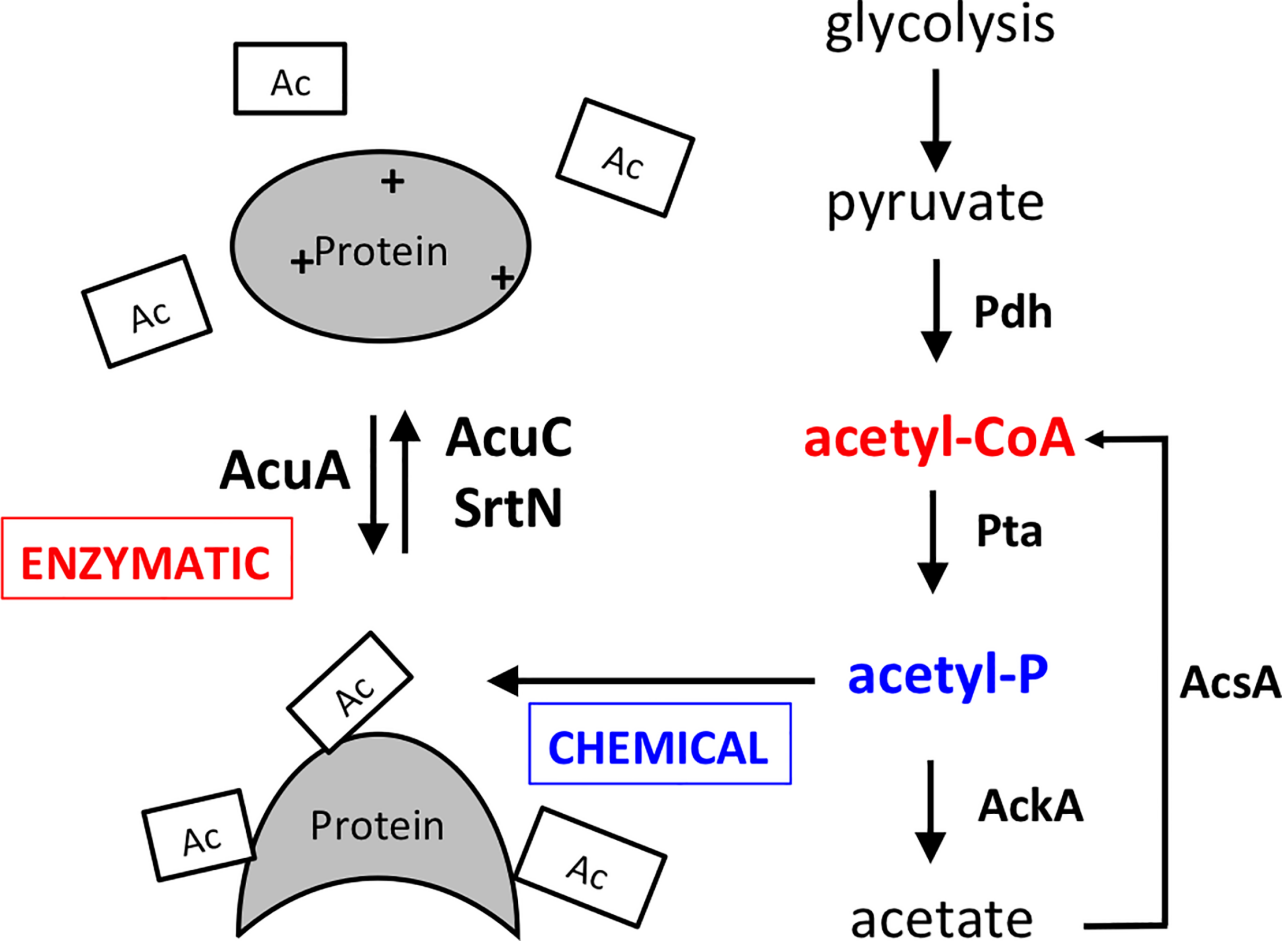
Protein lysine acetylation is carried out by either enzymatic or chemical mechanism in *B*. *subtilis*. A model of enzymatic and chemical acetylations on the lysine residues of a putative protein substrate. For the enzymatic mechanism, an acetyltransferase (AcuA in *B*. *subtilis*) takes the acetyl group (Ac) from a donor such as acetyl-CoA and adds it to a lysine residue on a target protein. This reaction can be reversed by the activity of a deacetyltransferase (AcuC or SrtN in *B*. *subtilis*). Chemical acetylation uses acetyl-phosphate, an intermediate in the acetate metabolic pathway, as the donor of the acetyl group to directly acetylate lysine residues of the target protein. “+” represents the positively charged lysine residue on the target protein. Pdh, pyruvate dehydrogenase; Pta, phosphotransacetylase; AckA, acetate kinase; AcsA, acetyl-CoA synthetase.

Post-translational modification enables bacteria to adapt quickly to changing environments. Instead of spending time and energy to express genes and translate new proteins, cells can quickly modify an existing protein to alter its activity (e.g. from inactive to active) in response to environmental changes [1, 2]. Protein lysine acetylation is a post-translational modification that involves the transfer of an acetyl group from a donor metabolite (e.g. acetyl-CoA or acetyl-phosphate) to a lysine residue of a protein [3]. Protein lysine acetylation can be achieved by two distinct mechanisms: chemical acetylation using acetyl-phosphate as a donor of the acetyl group, and enzymatic acetylation by lysine acetyltransferases using acetyl-CoA as the acetyl group donor [3, 4](Fig 1). During protein lysine acetylation, when a negatively charged acetyl group is covalently attached to a positively charged lysine residue, it can cause a change in charge and/or conformation of the protein, which may subsequently alter the protein activity [1, 4]. Protein lysine acetylation is a reversible process. Lysine deacetyltransferases are responsible for removing the acetyl group from the acetylated lysine residues of a protein. Originally characterized in eukaryotes, such as in the regulation of chromatin organization [3], protein lysine acetylation has now been recognized as an important post-translational modification in bacteria as well (reviewed in [4, 5]). Global acetylome analyses in bacteria have shown that protein lysine acetylation can occur on proteins involved in a variety of different functions such as central metabolism, transcription, DNA binding, motility, and cell size regulation [4, 6–10].

Biofilms are bacterial multicellular communities, in which individual cells stick with each other and are encased within a self-produced matrix composed of polysaccharides, proteins, and sometimes extracellular DNA providing a protective layer and a beneficial environment for the bacterial communities [11, 12]. Biofilms enhance cell to cell communication and virulence, rendering them a leading cause of hospital-acquired infections [13]. For example, in human opportunistic pathogens, such as *Pseudomonas aeruginosa* and *Staphylococcus aureus*, cells within the biofilms show a drastic increase in antibiotic resistance (estimated to be 100–1000 fold higher) and pathogenesis during infection [14, 15]. *Bacillus subtilis* is a soil-dwelling, spore-forming nonpathogenic bacterium. *B*. *subtilis* has served as a model organism for single cell development and bacterial signaling for decades [16–18], and more recently, for studies of bacterial multicellular development, or biofilm formation [11, 19, 20]. As a model system for biofilm studies, the regulatory network and signaling mechanisms that govern biofilm development have been extensively studied in *B*. *subtilis* [11, 19, 20]. In *B*. *subtilis*, a biofilm master repressor SinR controls dozens of matrix genes for production of protein fibers (TasA) and exopolysaccharides (EPS), two major components of the biofilm matrix that allows individual cells to stick to each other within a biofilm [11, 21–24]. Various environmental, plant host, and metabolic signals are shown to stimulate or modulate biofilm development in *B*. *subtilis* [11, 25–31]. In a recent study, we showed that endogenous or exogenously added acetate, a short-chain fatty acid from fermentation, can stimulate biofilm formation in *B*. *subtilis* [25]. Acetate may mediate multiple pathways in biofilm induction, one of which, as shown in the published study, is to activate an acetate-responsive pathway of three pairs of holin-antiholin-like genes [25, 32]. We also speculated in that study that there might be other potential biofilm induction mechanisms mediated by acetate in *B*. *subtilis* [25].

Concerning global protein lysine acetylation in *B*. *subtilis*, so far, there are three published global acetylome studies carried out under different conditions in different wild type strains [10, 33, 34]. One study demonstrated that adding glucose to the media increases global acetylation levels in *B*. *subtilis*, while another showed increased overall acetylation levels in exponential phase of the cells compared to those in stationary phase [33, 34]. In that study, it was further shown that acetylation of a specific cytoskeleton protein MreB, which plays an important role in cell shape regulation, can alter its protein function in *B*. *subtilis* [34]. Here, we were interested in understanding if global lysine acetylation plays an important role in bacterial multicellular processes, such as biofilm formation and swarming motility, in *B*. *subtilis*. We assayed a global acetylome from *B*. *subtilis* grown under biofilm-inducing conditions, investigated the effect of global lysine acetylation gene mutations on swarming and biofilm formation, and explored two individual proteins shown to be acetylated under biofilm inducing conditions. Our work revealed an important regulatory function of global lysine acetylation in *B*. *subtilis* multicellularity.

## Materials and methods

### Bacterial strains and media

A list of strains, plasmids, and oligonucleotides used in this study are included in Table 1. *B*. *subtilis* strain NCIB 3610 [19] and derived strains were routinely cultured in lysogenic broth (LB) (10 g tryptone, 5 g yeast extract, and 5 g NaCl per liter broth) at 37°C. Biofilm formation was induced in *B*. *subtilis* using LBGM (LB supplemented with 1% glycerol (v/v) and 100 μM MnSO_4_) [29] or MSgg minimal medium [19]. Enzymes were purchased from New England Biolabs (Ipswich, MA, USA). Chemicals and reagents were purchased from Sigma-Aldrich (St. Louis, MO, USA) or Fisher Scientific (Agawam, MA, USA). Oligonucleotides were purchased from Integrated DNA Technologies (San Jose, CA, USA) or Eurofins Genomics (Louisville, KY, USA). DNA sequencing was performed at Genewiz (Cambridge, MA, USA) or Eurofins Genomics. Antibiotics, as needed, were applied at the following concentrations: 1μg/ml of erythromycin, 100μg/ml of spectinomycin, 50μg/ml of kanamycin, and 10μg/ml of chloramphenicol for *B*. *subtilis*.

**Table 1:** Strains, plasmids, and oligonucleotides used in this study.

Strains	Description	Reference
PY79	a laboratory strain of *B*. *subtilis* used for genetic manipulation	[63]
168	a domesticated strain of *B*. *subtilis*	[64]
NCIB3610	an undomesticated strain of *B*. *subtilis*, capable of forming biofilms	[19]
DH5α	*E*. *coli* strain for molecular cloning	Invitrogen
ABR36	*acuA*::marker-less in 3610	this study
ABR69	*pta*::erm, *acuA*::marker-less in 3610	this study
ABR84	*ymcA*::spec, *amyE*::*PymcA-ymcA*, cm in 3610	this study
ABR 94	*ymcA*::spec, *amyE*::*PymcA-ymcA(K41R)*, cm in 3610	this study
ABR95	*ymcA*::spec, *amyE*::*PymcA-ymcA(K64R)*, cm in 3610	this study
ABR96	*ymcA*::spec, *amyE*::*PymcA-ymcA(K133R)*, cm in 3610	this study
ABR97	*ymcA*::spec, *amyE*::*PymcA-ymcA(K41R*, *K64R*, *K133R)*, cm in 3610	this study
ABR111	*gtaB*::kan, *amyE*::*PgtaB-gtaB(K83R)*, cm in 3610	this study
ABR112	*gtaB*::kan, *amyE*::*PgtaB-gtaB(K89R)*, cm in 3610	this study
ABR113	*gtaB*::kan, *amyE*::*gtaB(K89R*, *K191R)*, cm in 3610	this study
ABR114	*gtaB*::kan, *amyE*::*PgtaB-gtaB*, cm in 3610	this study
ABR115	*gtaB*::kan, *amyE*::*PgtaB-gtaB(K191R)*, cm in 3610	this study
ABR 116	*gtaB*::kan, *amyE*::*PgtaB-gtaB(K81R)*, cm in 3610	this study
ABR117	*gtaB*::kan, *amyE*::*PgtaB-gtaB(K83R*, *K89R*, *K191R)*, cm in 3610	this study
BKE29690	*acuA*::erm in 168	BGSC
BKE29710	*acuC*::erm in 168	BGSC
BKE09650	*srtN*:: erm in 168	BGSC
CY258	*motA*::erm in 3610	this study
KG007	*ackA*::erm in 3610	[25]
KG011	*pta*::erm in 3610	[25]
KG013	*pta*::erm, *amyE*::*PywbH-lacZ* in 3610	[25]
KG165	*acuA*::erm in 3610	this study
KG166	*acuC*::erm in 3610	this study
KG167	*srtN*::erm in 3610	this study
RL4619	*ymcA*::spec in 3610	[54]
YC876	*gtaB*::kan in 3610	this study
YY388	*acuC*::marker-less, *srtN*::marker-less in 3610	this study
**Plasmids**		
pABR77	pDG1662, *amyE*::*PymcA-ymcA(K41R)*, amp, cm	this study
pABR78A	pDG1662, *amyE*::*PymcA-ymcA(K64R)*, amp, cm	this study
pABR78B	pDG1662, *amyE*::*PymcA-ymcA(K133R)*, amp, cm	this study
pABR79	pDG1662, *amyE*::*PymcA-ymcA(K41R*, *K64R*, *K133R)*, amp, cm	this study
pEH89	pDG1662, *amyE*::*PgtaB-gtaB*, amp, cm	this study
pEH90	pDG1662, *amyE*::*gtaB(K81R)*, amp, cm	this study
pEH91	pDG1662, *amyE*::*PgtaB-gtaB(K83R)*, amp, cm	this study
pEH92	pDG1662, *amyE*::*PgtaB-gtaB(K89R*), amp, cm	this study
pEH93	pDG1662, *amyE*::*PgtaB-gtaB(K191R)*, amp, cm	this study
pEH94	pDG1662, *amyE*::*gtaB(K89R*, *K191R)*, amp, cm	this study
pEH95	pDG1662, *amyE*::*gtaB(K83R*, *K89R*, *K191R)*, amp, cm	this study
pDG1662	*B*. *subtilis amyE* insertional vector	BGSC
pDR244	cre+TS origin loops out erm resistant marker using cre and temperature sensitive origin	BGSC
pYC107	pDG1662, *amyE*::*PymcA-ymcA*, amp, cm	this study
**Primers**		
acuA-F	5’ ctcaatttttaaaatataaaccatgttcaaaacgct 3’	
acuA-R	5’ attgctgtttcaagcgtatccgtc 3’	
acuC-F	5’ ttgtcttccgtgtaaaaacgatgaa 3’	
acuC-R	5’ gtaaaggataacaagacaaatgaacac 3’	
gtaB-F	5’ gtacgaattccttgatcgcttcaggcctggtcc 3’	
gtaB-R	5’ gtacggatcccgcagttgataatgaagagcatacattgactttgat 3’	
gtaB-(K81R)-F	5’ aacctagaagaaagaggaaaaactgag 3’	
gtaB-(K81R)-R	5’ ctcagtttttcctctttcttctaggtt 3’	
gtaB-(K83R)-F	5’ gaagaaaaaggaagaactgagctgctt 3’	
gtaB-(K83R)-R	5’ aagcagctcagttcttcctttttcttc 3’	
gtaB-(K89R)-F	5’ gagctgcttgaaagagtgaaaaaggct 3’	
gtaB-(K89R)-R	5’ agcctttttcactctttcaagcagctc 3’	
gtaB-(K191R)-F	5’ aacttcgttgaaagaccgcctaaaggc 3’	
gtaB-(K191R)-R	5’ gcctttaggcggtctttcaacgaagtt 3’	
srtN-F	5’ tctaaataaagaggaaaaggaacgggc 3’	
srtN-R	5’ taatcaataaattaaaagaaaaagctattccttcg 3’	
ymcA-(K41R)-F	5’ aatgagaatgacagagtgtccacaatc 3’	
ymcA-(K41R)-R	5’ gattgtggacactctgtcattctcatt 3’	
ymcA-(K64R)-F	5’ aagcattatgaaaggcatgaagcgctc 3’	
ymcA-(K64R)-R	5’ gagcgcttcatgcctttcataatgctt 3’	
ymcA-(K133R)-F	5’ gaaaccggttcaagggtgaagcattca 3’	
ymcA-(K133R)-R	5’ tgaatgcttcacccttgaaccggtttc 3’	

* Underlined sequences indicate lysine codons being substituted by arginine codons.

### Bacterial strain construction

The *acuA*::*erm* (BKE29690), *acuC*::*erm* (BKE29710), and *srtN*::*erm* (BKE09650) insertional deletion mutants in the *B*. *subtilis* 168 background were purchased from the *Bacillus* Genetic Stock Center (BGSC, http://www.bgsc.org) and DNA fragments containing the insertional deletion were introduced into 3610 via SPP1 phage-mediated general transduction [35] to generate strains KG165, KG166, and KG167, respectively. The construction of *pta*::*erm* (KG011) and *ackA*::*erm* (KG007) deletion mutants of 3610 was described in a previous study [25]. To allow combination of the Δ*acuA* deletion mutation with *pta*::*erm*, a marker-less *acuA* deletion mutant (ABR36) was first constructed using a temperature-sensitive, *cre*-bearing vector (pDR244) (BGSC). The genomic DNA containing *pta*::*erm* was then prepared and introduced into ABR36 using SPP1 phage-mediated general transduction. Colonies were selected for on an LB agar plate supplemented with erythromycin. *acuA* insertional deletion was verified by PCR using primers acuA-F and acuA-R (Table 1). To construct the double marker-less deletion mutant of Δ*acuC*Δ*srtN* (YY388), the *erm* marker was first removed from each mutant using the *cre* temperature-sensitive vector pDR244. The genomic DNA of Δ*acuC* was then introduced into Δ*srtN* marker-less deletion mutant. The resulting mutant YY388 were confirmed for the double deletion mutations by PCR using primers acuC-F and acuC-R, and srtN-F and srtN-R. Transformants were selected on LB agar plates supplemented with erythromycin and the presence of both Δ*acuC* and Δ*srtN* was verified by PCR using primers acuC-F and acuC-R, and srtN-F and srtN-R [36].

Site-directed mutagenesis on selected lysine codons in *ymcA* and *gtaB* was performed by adapting a Phusion Site-Directed Mutagenesis kit (Thermo Fisher Scientific) and the published protocol. Plasmids pYC107 and pEH089 were used as templates for *ymcA* and *gtaB*, respectively. Briefly, pEH089 was constructed using the *amyE* integration vector, pDG1662 (BGSC). The *gtaB* promoter and gene were amplified by PCR using primers gtaB-F and gtaB-R and 3610 genomic DNA as the template. The PCR product and pDG1662 plasmid were purified and double digested with EcoRI and BamHI. After digestion, the PCR product and plasmid DNA were ligated using T4 ligase. The ligation product was then transformed into *E*. *coli* DH5α by chemical transformation. The recombinant plasmid was prepared from *E*. *coli* cells, and verified by DNA sequencing. For site-directed mutagenesis of *ymcA* and *gtaB*, the plasmids were methylated by following the manufacturer’s protocol (Thermo Fisher Scientific). Briefly, CpG methyltransferase (M.SssI) was incubated with the plasmid using S-adenosylmethionine (SAM) as a cofactor. This added a methyl group to the C5 position of all cytosine nucleotides on the plasmid. Multiple pairs of overlapping primers gtaB-(K81R)-F, gtaB-(K81R)-R, gtaB-(K83R)-F, gtaB-(K83R)-R, gtaB-(K89R)-F, gtaB-(K89R)-R, gtaB-(K191R)-F, gtaB-(K191R)-R, ymcA-(K41R)-F, ymcA-(K41R)-R, ymcA-(K64R)-F, ymcA-(K64R)-R, ymcA-(K133R)-F, ymcA-(K133R)-R) designed to mutate a lysine codon to an arginine codon were used to site-specifically mutate the wild type allele on the template plasmid. The resulting mutated plasmid was introduced into *E*. *coli* DH5α by chemical transformation. Plasmids were isolated using DNA mini-prep kits (Qiagen) and the presence of mutations was verified by DNA sequencing. Mutated plasmids were then introduced by transformation into the *B*. *subtilis* laboratory strain PY79. Transformants were selected for double-crossover recombination at the chromosomal *amyE* locus on LB agar plates with appropriate antibiotics and by verification of loss of amylase activities on LB plus starch plates. The resulting complementation constructs were then introduced into their respective deletion strains of either Δ*ymcA* (RL4619) or Δ*gtaB* (YC876) to generate *ymcA* mutant alleles (ABR85, ABR96-97) and *gtaB* mutant alleles (ABR111-117) by using SPP1 phage-mediated general transduction. Transductants were verified by selecting on LB plus appropriate antibiotics, starch amylase test, and DNA sequencing for the presence of the site-directed codon substitutions.

### Generation of global acetylome

#### Protein extraction

*B*. *subtilis* strains 3610, KG007(*pta*::*erm*) and KG165(*acuA*::*erm*) were grown to stationary phase in shaking LBGM medium at 37°C. The culture was harvested by centrifugation and the cell pellet was grinded by liquid nitrogen and transferred to a 5-mL centrifuge tube. The cell powder was sonicated three times on ice using high intensity ultrasonic processor (Scientz) in a lysis buffer [8 M urea, 1% Triton-100 (v/v), 65 mM dithiothreitol (DTT) and 0.1% Protease Inhibitor Cocktail (w/v)]. The remaining cell debris was removed by centrifugation at 20,000 g at 4°C for 10 min. Finally, the protein was precipitated with cold 15% trichloroacetic acid (v/v) for 2 hours at -20°C. After centrifugation at 4°C for 10 min, the supernatant was discarded. The remaining precipitate was washed with cold acetone three times. The protein was dissolved in buffer [8 M urea, 100 mM triethylammonium bicarbonate (TEAB), pH 8.0], and the protein concentration was determined using a 2-D Quant kit according to the manufacturer’s instructions (GE Healthcare).

#### Trypsin digestion

The extracted protein solution was reduced with 10 mM (DTT) for 1 hr at 37°C and alkylated with 20 mM iodoacetamide for 45 min at room temperature in darkness. For trypsin digestion, the protein sample was diluted by adding 100 mM TEAB to urea at the concentration less than 2 M. Finally, trypsin was added to the sample at a 1:50 trypsin-to-protein mass ratio for the first overnight digestion and then a 1:100 trypsin-to-protein mass ratio for a second round of 4 hr digestion.

#### Tandem mass tag (TMT) labeling

After trypsin digestion, the peptides were desalted by Strata X C18 SPE column (Phenomenex) and vacuum-dried. The peptide sample was reconstituted in 0.5 M TEAB and processed according to the 6-plex TMT kit and the manufacturer’s protocol. Briefly, one unit of TMT reagent (defined as the amount of reagent required to label 1.25 mg of protein) was thawed and reconstituted in acetonitrile. The peptide mixtures were combined and then incubated for 2 hrs at room temperature, desalted, and then dried by vacuum centrifugation.

#### HPLC fractionation

The peptide sample was then fractionated by high pH reverse-phase HPLC using an Agilent 300Extend C18 column (5 μm particles, 10 mm ID, 250 mm length). Briefly, peptides were first separated into 80 fractions with a gradient of 2% to 60% acetonitrile (v/v) in 10 mM ammonium bicarbonate pH 10 over 80 min. The peptides were then combined into 8 fractions and dried by vacuum centrifuging.

#### Affinity enrichment

To enrich for acetylated lysine residues on the peptides, tryptic peptides dissolved in NETN buffer (100 mM NaCl, 1 mM EDTA, 50 mM Tris-HCl, 0.5% NP-40 (v/v), pH 8.0) were incubated with pre-washed anti-acetyl-lysine antibody-bound beads at 4°C overnight with gentle shaking. The beads were washed four times with NETN buffer and twice with ddH2O. The bound peptides were eluted from the beads with 0.1% trifluoroacetic acid (v/v). The eluted fractions were combined and vacuum-dried. The resulting peptides were cleaned with C18 ZipTips (Millipore) according to the manufacturer’s instructions, followed by LC-MS/MS analysis.

#### LC-MS/MS analysis

The enriched peptides were dissolved in 0.1% formic acid (v/v) and directly loaded onto a reversed-phase pre-column (Acclaim PepMap 100, Thermo Scientific). Peptide separation was performed using a reversed-phase analytical column (Acclaim PepMap RSLC, Thermo Scientific). The gradient was comprised of an increase from 6% to 23% solvent B [0.1% formic acid (v/v) in 98% acetonitrile (v/v)] for 24 min, 23% to 35% for 8 min and climbing to 80% in 4 min then holding at 80% for the last 4 min, all at a constant flow rate of 280 nl/min on an EASY-nLC 1000 UPLC system. The resulting peptides were analyzed by Q Exactive^TM^ Plus hybrid quadrupole-Orbitrap mass spectrometer (Thermo Fisher Scientific).

### Acetylome analyses and statistics

#### Relative ratio comparison

Overall acetylation levels between wild type 3610 and *pta*::*erm* and *acuA*::*erm* were compared by the use of the calculated ratio of relative intensity. Any value above 1.0 was considered an increase and any value below 1.0 was considered a decrease relative to wild type. Before further analysis, peptides that did not have a ratio were removed from the list. Outliers were also removed as determined by a scatter plot correlation of two 3610 replicates. Cut offs were arbitrarily determined to be less than 0.5 and greater than 1.7 for 3610-A and greater than 2.0 for 3610-B. A second round of outliers were removed using Gibbs Outlier Test. The outlier test was used for the peptide ratio set and the protein ratio set. The acetylation relative ratio values were calculated at the protein level by taking the average of multiple peptides. Heat maps were generated using Prism7.

#### Protein classification

Proteins were annotated using Gene Ontology (GO) bioinformatics derived from the Unit-Prot-GOA database (www.http://www.ebi.ac.uk/GOA/). An identified protein was matched to its UniProt ID, which was then mapped to the GO ID. If a protein was not found in UniProt, then InterProScan software was used to annotate proteins based on InterPro database domain identification (http://www.ebi.ac.uk/interpro/).

Once proteins were categorized by biological function, relative intensity ratio of each biological process was calculated by taking the average of all proteins annotated within each category.

#### Motif analysis

Peptides were analyzed for residue motif six amino acids upstream and downstream of acetylated lysine residue using Motif-X software. The minimum number of peptides that could occur in one motif was set at 20 peptides. The motif analysis statistics test significance threshold for binomial probability was set at 0.0000001. This value was selected in order to maintain a low false positive rate.

### Swarming assay

The swarming assay was performed following a modified protocol [37]. Briefly, cells were grown in shaking in LB at 37°C to about OD_600_ = 1.0 and concentrated about 10 fold (to OD_600_ about 10). 10 μL of concentrated cells were spotted onto the center of a LB plate solidified with 0.7% agar (w/v) and previously dried under flow hood for 10 minutes. Plates were dried for another 10 minutes and then placed in a 37°C incubator. After 2 hrs of incubation, the swarm radius was measured every hour along the same axis for a period of 7 hours. The final swarm radius was measured the next day after 24 hrs from initial plating. Assays were done in triplicate. Error bars represent values of standard deviations.

### Biofilm assay

For colony biofilm formation, cells were grown to exponential phase in LB broth and 2-μl of the culture was spotted onto LBGM [29] or MSgg [19] plates solidified with 1.5% agar (w/v). LBGM plates were incubated at 30°C for about 72 hrs and MSgg plates were incubated at 25°C for about 120 hrs. For pellicle biofilm formation, cells were grown to exponential phase in LB broth, and 2-μl of the culture was inoculated into 2-ml of LBGM or MSgg broth in a 24-well microtiter plate (Corning, NY, USA). LBGM pellicles were incubated for about 72 hrs at 30°C and MSgg pellicles were incubated for 48 hrs at 25°C. Images of colony and pellicle biofilms were taken using a Leica MSV269 dissecting scope or a Nikon Coolpix camera.

### Biofilm biomass assay

To measure the biofilm biomass, the colony or pellicle biofilm was grown as described above. After 72 hrs of growth at 30°C, the biofilm was harvested and placed into a 2-mL microcentrifuge tube. Any residual media was removed from the colony/pellicle matrices after centrifugation, samples were washed with PBS buffer, and tubes were set to air-dry in a flow hood overnight. The biomass was then weighed on a scale to quantify the biofilm produced. All assays were done in triplicate.

## Results

### Analyses of global acetylomes in wild type *B*. *subtilis* and two putative acetylation mutants

We generated a global acetylome of *B*. *subtilis* (the original raw data was provided in S1 Table) by applying a method similar to previously published studies [10, 38]. Briefly, we cultured wild type *B*. *subtilis* NCIB3610 (abbreviated as 3610 hereafter) cells in LBGM [LB supplemented with 1% glycerol (v/v) and 100 μM manganese], a biofilm-inducing medium [29]. After grown to stationary phase in which cells reached an optical density of about O.D. _600_ = 3.0, cells were harvested and lysed, and the lysate was enriched for acetylated proteins using an anti-acetylated lysine antibody (Abcam, USA). The anti-acetylated lysine antibody has been used in numerous similar studies previously. Although some studies raised caution about the binding specificity and affinity when using commercially available polycolonal anti-acetylated lysine antibodies, our own western blot analysis demonstrated fairly robust signals using the total protein lysate (data not shown), indicating relative effectiveness of the antibody. Mass spectrometry was then performed to identify peptides with acetylated lysine residues. Subsequent acetylome analyses allowed us to identify a total of 1772 lysine acetylation sites in 826 different proteins, which covers about 19.6% of all proteins encoded by *B*. *subtilis* genome (S1 Table). Among the 826 proteins, about 50% showed only one acetylated lysine residue in the candidate protein (S1 Fig).

**Fig 2:**
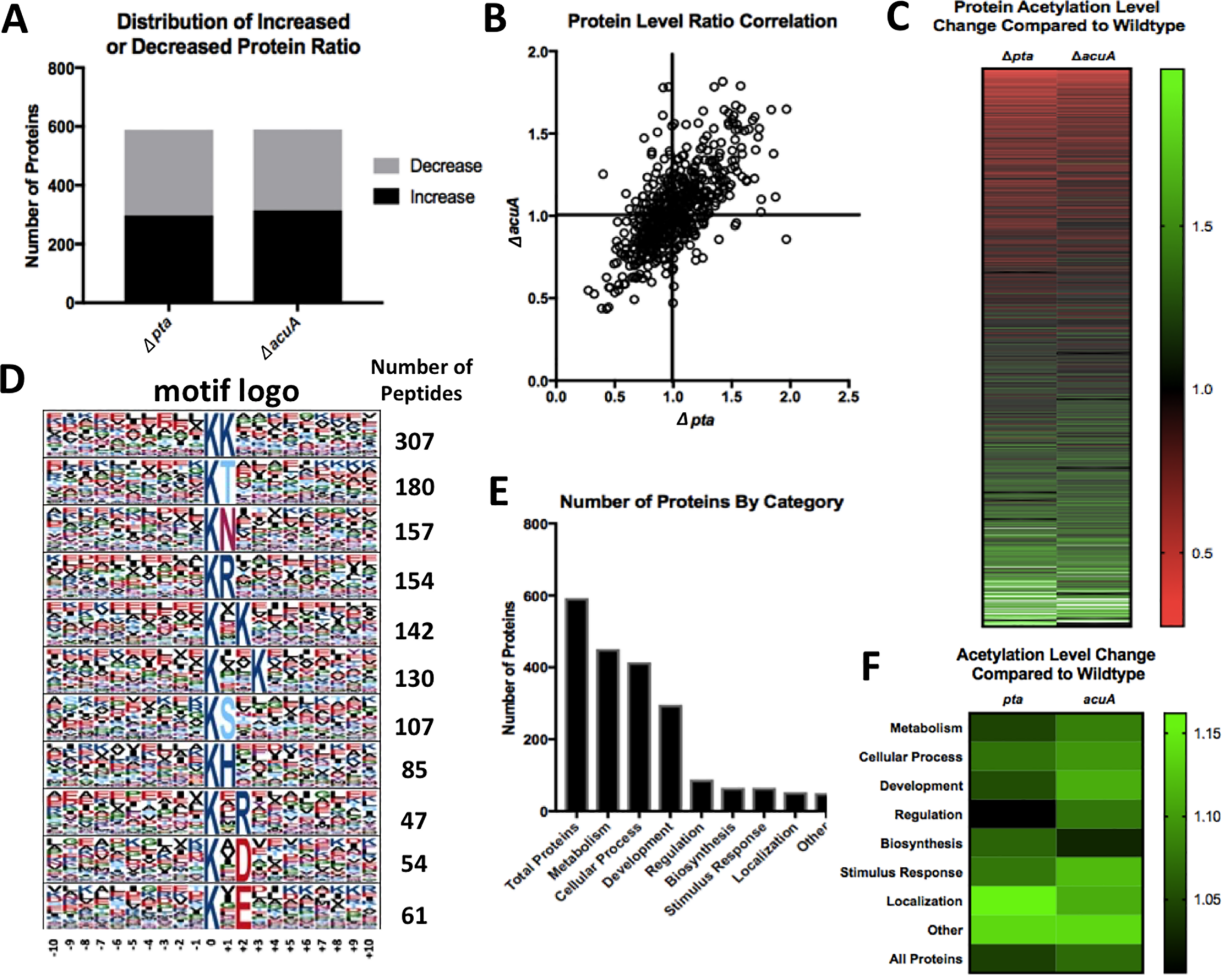
*B*. *subtilis* global acetylome analyses. **(A)** Distribution of proteins with increased or decreased acetylation levels in either the Δ*pta* or Δ*acuA* mutant when comparing to the wild type. About 50% (n = 590) of the acetylated proteins had a mild increase in the acetylation level in either one of the two mutants while the other 50% (n = 590) had a mild decrease. **(B)** Analysis of acetylated proteins by comparing relative intensity ratios of the acetylation signals in Δ*pta* or Δ*acuA* to the wild type. Correlation of r = 0.66 (n = 576). The majority of proteins in the mutants demonstrated no significant change in the acetylation level compared to the wild type. **(C)** Heat map showing the change in acetylation levels of all acetylated proteins between the wild type to the respective mutants (n = 585). Green indicates an increase while red indicates a decrease in the acetylation level. **(D)** Analysis of acetylated peptides identified specific acetylation motifs associated with lysine acetylation. Acetylated lysine residues tend to be flanked by charged residues such as glutamic acid. (**E)** Acetylated proteins can be organized into functional categories. The majority of acetylated proteins are involved in cellular metabolism and/or developmental processes. **(F)** Heatmap demonstrating fold changes in the acetylation level by protein category when comparing the two mutants to the wild type.

In addition to identifying global lysine acetylation in the wild type cells, we also characterized global acetylomes from two single deletion mutants of *B*. *subtilis* (Δ*pta* and Δ*acuA*). The *pta* gene encodes a phosphotransacetylase that converts acetyl-CoA to acetyl-phosphate (Fig 1)[6, 33, 39]. A strain lacking the *pta* gene (Δ*pta*) is expected to have decreased production of acetyl-phosphate, leading to impaired chemical acetylation. The *acuA* gene encodes a lysine acetyltransferase in *B*. *subtilis* whose activity was experimentally confirmed [40]. A strain lacking *acuA* (Δ*acuA*) is expected to be impaired in enzymatic acetylation. For direct comparison of the global acetylomes from the wild type and the two deletion mutants, the ratio of the relative intensity of the acetylation peaks corresponding to the same proteins in different acetylomes was calculated. A ratio above 1.0 indicated an increase in acetylation in a specific protein in the mutant compared to the wild type, while a ratio below 1.0 suggested a decrease. About 30% of the acetylated proteins did not have a ratio, suggesting that the level of the acetylated proteins was below the detection limit in one or both mutants. Surprisingly, analysis of acetylation levels between the wild type, Δ*pta*, and Δ*acuA* demonstrated neither a significant increase nor a decrease in the global acetylation level (Fig 2A–2C). Further, comparison between Δ*acuA* and Δ*pta* did not show a significant difference either in lysine acetylation level as seen by correlation of r = 0.64 (Fig 2B). Although there was little quantitative difference in lysine acetylation at the global level between the wild type and the mutants, individual proteins showed differential acetylation levels or different acetylation patterns (acetylation occurs on different lysine residues in the same protein) (Fig 2A and 2C). Within the 70% of the acetylated proteins characterized in the acetylomes of both the wild type and the mutants, about half of them showed a mild increase in the acetylation level while the other half showed a mild decrease (Fig 2A). In terms of different acetylation patterns, presumably, chemical or enzymatic acetylation mechanism may target different lysine residues in the same protein for modification.

### Identification of specific lysine acetylation motifs and functional clusterings of acetylated proteins in the global acetylome

Analysis of the acetylome also revealed the presence of several putative lysine acetylation motifs on the modified peptides. The most common motif is two lysine residues in tandem (observed in 579 out of 1424 acetylated peptides, 40.7%, Fig 2D). Further, the presence of multiple glutamic acid residues in immediate upstream of the acetylated lysine is apparent. This is consistent with a previous study, in which it was suggested that the negatively charged glutamic acid serves as a catalyst to increase the reactivity of the lysine residue with the acetyl group [4]. Similar glutamic acid-rich motifs for lysine acetylation have been identified in the analyses of other *B*. *subtilis* global acetylomes as well as in Gcn5-related acetyltransferase- and acetyl-phosphate-mediated reactions in higher organisms [4].

In addition to motif analysis, we also classified the acetylated proteins into various biological function groups based on Gene Ontology Annotation derived from the UniProt-GOA database (Fig 2E)[41]. The majority of the acetylated proteins is associated with cellular and metabolic processes, which is expected since protein lysine acetylation was previously shown to be intimately linked to central metabolism [42, 43]. Comparing the relative intensity of the functional groups between the two mutants and the wild type demonstrated a mildly lower overall intensity ratio for the global protein acetylation in Δ*pta* than in Δ*acuA* (Fig 2C and 2F). This might support the idea that chemical acetylation plays a bigger role in global protein lysine acetylation than the enzymatic pathway under our tested conditions. Taken together, our data demonstrated the presence of specific acetylation motifs during protein lysine acetylation and that acetylated proteins fall into distinct functional groups beyond cellular and metabolic processes, which have been primarily investigated in previous studies on bacterial protein lysine acetylation [4, 42].

### Global protein lysine acetylation mutants are impaired in swarming motility

**Fig 3:**
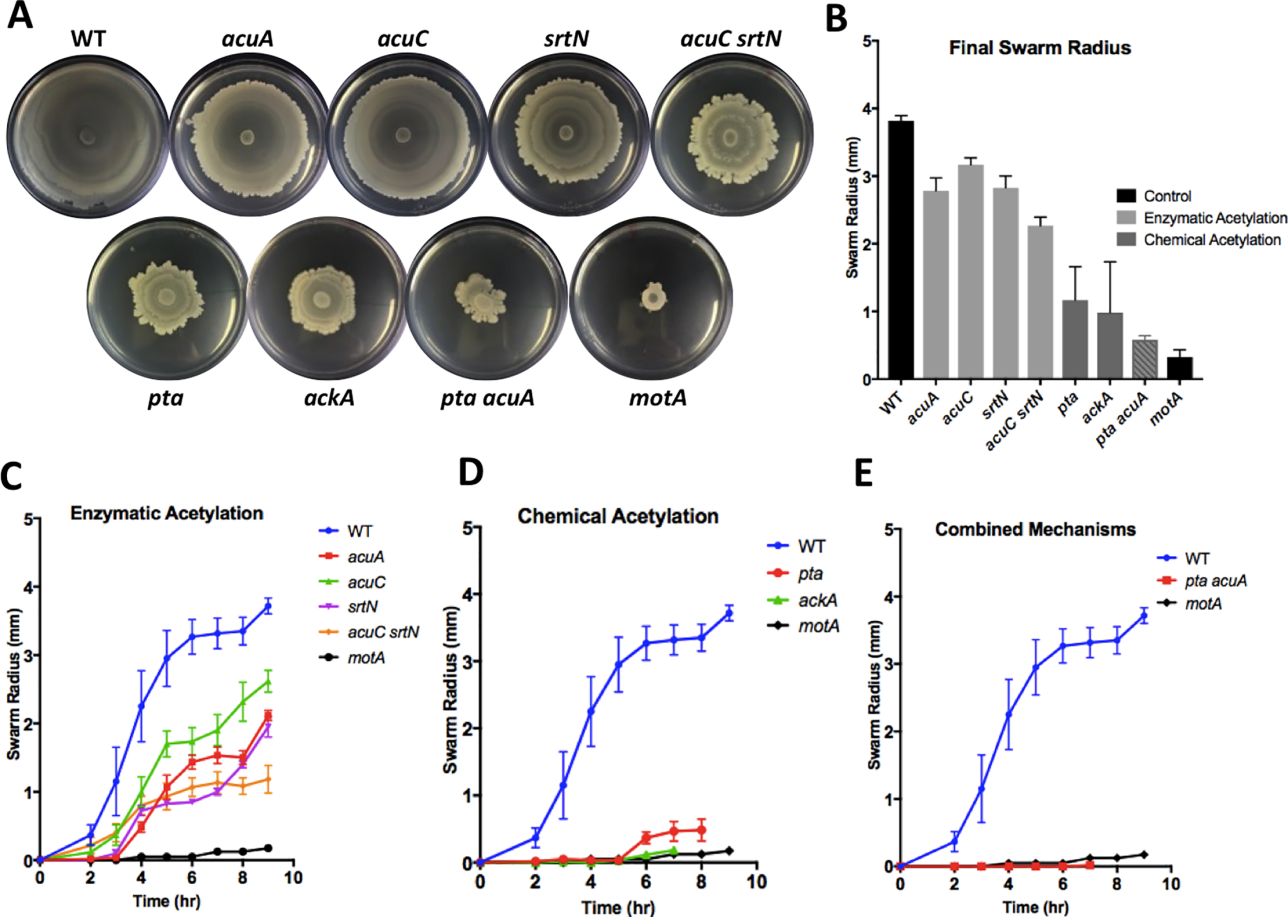
Lysine acetylation mutants of *B*. *subtilis* showed impaired swarming motility. **(A)** Images of the swarming plates by the wild type and various acetylation mutants after 20 hours of incubation at 37°C. All figures are representatives of multiple experiments and statistics were calculated using standard error of the mean (n = 3). T-test significance calculated as p<0.05. **(B-D)** Various acetylation mutants as shown were plated on 0.7% LB agar (w/v) and the swarming radius was measured along the same axis over time. Δ*motA* was used as a negative control (n = 3). **(B)** The enzymatic acetylation mutants (Δ*acuA*, Δ*acuC*, Δ*srtN*, and Δ*acuC*Δ*srtN*) had slower swarming kinetics than the wild type after 9 hours of incubation (p<0.0002). **(C)** The chemical acetylation mutants (Δ*pta* and Δ*ackA)* demonstrated a severe swarming defect compared to the wild type (p<0.0001). **(D)** The double mutant of Δ*pta*Δ*acuA* demonstrated a significant swarming defect compared to the wild type (p<0.0001). Δ*pta*Δ*acuA* was not significantly different from Δ*motA* (p>0.35). **(E)** The final swarm radius of the acetylation mutants after 20 hrs of incubation (n = 3). The swarming kinetics of the acetylation mutants were all significantly different from that of the wild type (p<0.0004) except for Δ*acuC* and Δ*srtN* (p>0.07).

In our characterized acetylome, a significant number of proteins potentially involved in general motility and two proteins (SwrAA and SwrC) known to be specifically involved in multicellular swarming in *B*. *subtilis* were found to be acetylated (Table 2). Based on this information, we speculated that protein lysine acetylation could play a role in regulating the activity of those proteins and thus swarming motility. To test that, we constructed several mutants presumably altered in either the global chemical or the enzymatic acetylation pathway, and compared swarming motility by those mutants to that of the wild type (Fig 3A). Swarming is the ability of cells to collectively expand outward on semi-solid surfaces as they search for more nutrients and surface area for colonization [44]. The single deletion mutants used in the swarming assay include Δ*acuA* and Δ*pta*, both of which are expected to be impaired in lysine acetylation as discussed above. Single deletion mutants used here also include Δ*acuC* and Δ*srtN*. In *B*. *subtilis*, *acuC* and *srtN* encode a lysine deacetylase and a sirtuin deacetylase, respectively, both of which were shown to be involved in lysine deacetylation (Fig 1) [45, 46]. The Δ*acuC* and Δ*srtN* single mutants are expected to demonstrate elevated acetylation levels due to reduced activities in deacetylation [33]. Lastly, *ackA* encodes an acetate kinase that converts acetyl-phosphate to acetate [47]. Deletion of *ackA* should lead to an accumulation of both acetyl-phosphate and acetyl-CoA, and subsequently elevated acetylation levels through both chemical and enzymatic pathways (Fig 1) [6, 33, 47, 48]. In addition to the 5 single deletion mutants, double mutants were also constructed. These deletion mutants are generally referred to as acetylation mutants in this study.

**Table 2:** A list of acetylated proteins involved in motility.

Protein	Function	Lysine Residues in Protein			Acetylated Lysine Residues	Reference
		Total Number of Lysines	% Lysines in Total Protein	% Acetylated Lysines out of all Lysines		
**DegS**	regulatory	31	8%	6%	259 223	[34] this study
**DegU**	regulatory	13	6%	15%	195 181	[34] this study
**FliD**	flagellar machinery	39	8%	3%	385	this study
**FliF**	flagellar machinery	44	8%	5%	9, 72	this study
**FliG**	flagellar machinery	19	6%	5%	31	this study
**FliH**	flagellar machinery	20	10%	5%	94	this study
**FliJ**	flagellar machinery	22	15%	9%	53, 46	this study
**FlgG**	flagellar machinery	13	5%	8%	35	this study
**Hag**	flagellar subunit	15	5%	33%	29, 51, 143 51, 135 25, 29, 51, 135, 136, 143	[34] [33] this study
**SwrAA**	regulatory	10	9%	10%	83	this study
**SwrC**	surfactin secretion	88	8%	7%	449 527, 535, 605, 783, 790	[34] this study

List of acetylated motility proteins found in our acetylome. Also listed is the percent lysine residue content in each protein, the percent of total lysine residues that were found acetylated, and the specific acetylated lysine sites. Included are acetylated lysine residues identified in other *B*. *subtilis* acetylome studies [10, 34, 38].

For swarming assays, cells were grown to mid-exponential phase (O.D._600_ = 1.0) and then spotted onto LB plates containing 0.7% agar (w/v). The swarming radius was periodically measured along the same axis. Swarming kinetics of the acetylation mutants were compared to that of the wild type and of Δ*motA*. The *motA* gene encodes a motor subunit essential for the flagella function [49]. The *motA* mutant was previously shown to be completely deficient in swarming motility and used as a negative control [50]. Interestingly, our results showed that there was a significant swarming defect in all the mutants tested when compared to the wild type (Fig 3A–3D). The enzymatic pathway mutants (Δ*acuA*, Δ*acuC*, and Δ*srtN*) and the double mutant (Δ*acuC*Δ*srtN*) all demonstrated a significantly slower rate of swarming than the wild type (Fig 3B). The chemical pathway mutants, Δ*pta* and Δ*ackA*, demonstrated an even more severe defect, similar to the negative control strain Δ*motA*, suggesting that chemical acetylation may play a bigger role in regulating swarming motility than the enzymatic acetylation (Fig 3C). Finally, the double mutant of Δ*pta*Δ*acuA* also demonstrated a very severe swarming defect (Fig 3D). A mild growth defect in both Δ*pta* and Δ*ackA* was observed, which could partially contribute to the swarming defect (S2 Fig), To evaluate the possible impact of slower growth rate on swarming, we extended the incubation time of the swarming plates from 10 hours to 24 hours. When comparing the final swarm radius after 24 hours of incubation, in all mutants, the swarming impairment persisted indicating a defect rather than a delay (Fig 3A and 3E). This observation supported the idea that the growth defect was not the primary factor for the defect in swarming (Fig 3A). In all, our data showed that global lysine acetylation is involved in regulating swarming activity in *B*. *subtilis*. It was somewhat surprising to us that both acetylation and deacetylation mutants showed a defect in swarming motility. One possibility could be that some individual proteins involved in motility need acetylation for activity while others may rely on deacetylation for their function. In future studies, it will be interesting to focus on individual proteins involved in motility. For example, Hag, the subunit of the flagella, is a protein in which multiple lysine residues were identified to be acetylated in this study as well as in previous studies (Table 2) [33, 34].

### Global protein lysine acetylation mutants are impaired in biofilm formation

**Fig 4:**
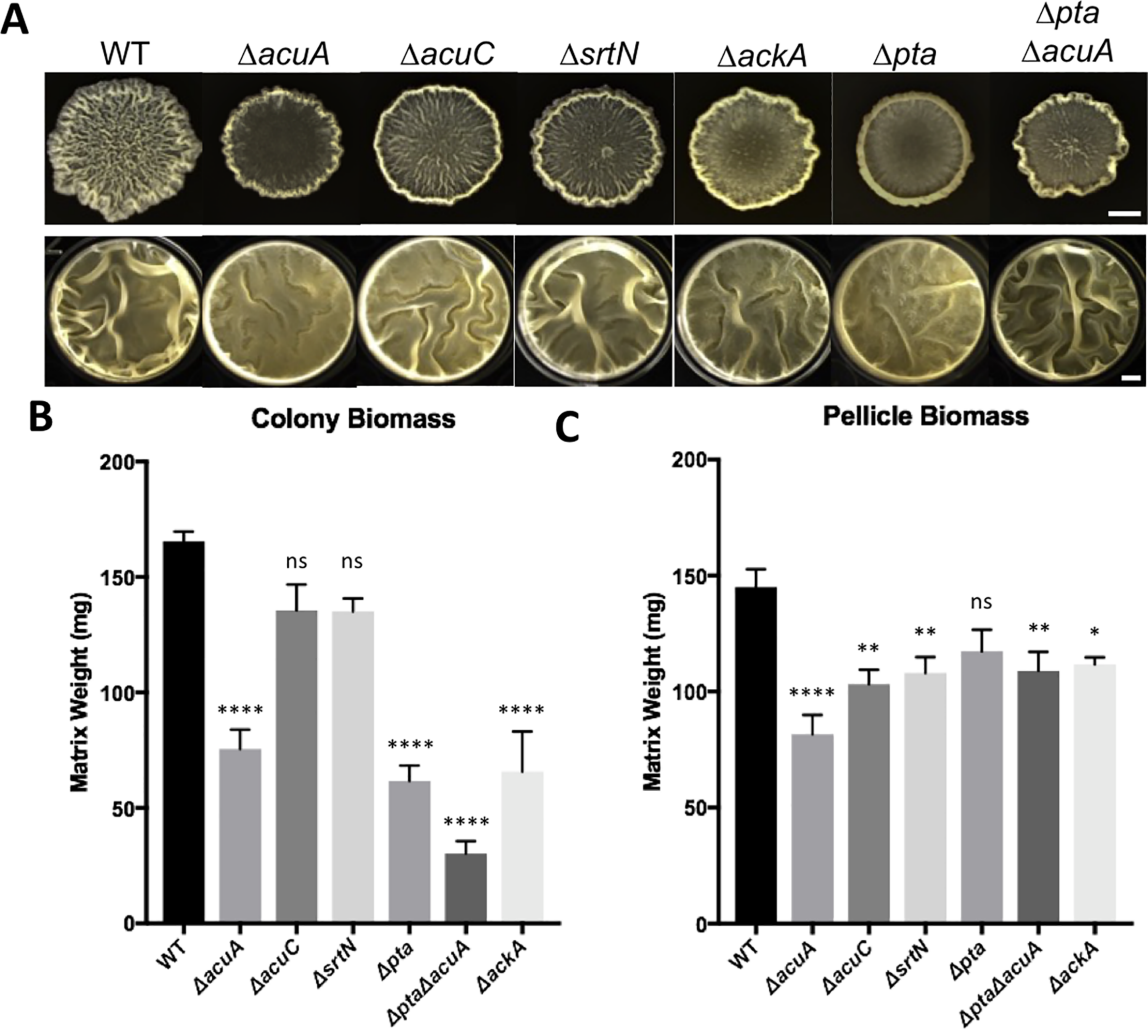
Lysine acetylation mutants are impacted for biofilm formation. **(A)** Colony and pellicle biofilm formation by various acetylation mutants on LBGM media. The scale bar in the upper panel, 5 mm; the scale bars in the lower panel, 200 μm. **(B and C)** Quantification of the biomass of colony **(B)** and pellicle **(C)** biofilms. Colony/pellicle biofilm was harvested, air-dried, and weighed after 72 hours of incubation. Assays were done in triplicate statistics were calculated using standard error of the mean (n = 8). T-test significance calculated as p<0.05.

Our acetylome analyses also identified a number of proteins previously shown to be important for biofilm formation in *B*. *subtilis* [11, 51], being acetylated at multiple lysine residues (Table 3), indicating potential effects of global lysine acetylation on the activities of those proteins and thus biofilm formation. To investigate that, we first performed biofilm assays using various acetylation mutants that we constructed and described above in the swarming assay. In biofilm assays, the presence of increased wrinkles serves as an indicator for increased biofilm robustness, while decrease in wrinkles or a flat biofilm indicates a biofilm defect [19]. If global lysine acetylation plays a positive role in biofilm formation, we should expect a biofilm defect for the mutants deficient in acetylation (e.g. Δ*acuA*, Δ*pta*, and Δ*pta*Δ*acuA*), and *vice versa* an increase in biofilm robustness for the mutants impaired in deacetylation (e.g. Δ*acuC* and Δ*srtN*). Surprisingly, all the mutants demonstrated a visual defect in colony biofilm formation (Fig 4A). Results from biomass quantitative analyses also support the above observations demonstrating a significant reduction in the biofilm biomass in Δ*acuA*, Δ*pta*, Δ*pta*Δ*acuA*, and Δ*ack* while no significant reduction was seen in Δ*acuC and* Δ*srtN* (Fig 4B). In pellicle biofilm formation, the phenotypes were much milder; only Δ*acuA* and Δ*pta* showed a modest defect (Fig 4A). This phenotype was also consistent with the results of biofilm biomass quantification (Fig 4C). Our results that both acetylation and deacetylation mutants showed a defect in biofilm formation were similar to what was observed previously in swarming assays (Fig 3). In general, our results support the idea that global protein lysine acetylation is involved in the regulation of biofilm formation in *B*. *subtilis*.

**Table 3:** A list of acetylated proteins involved in biofilm formation.

Protein	Function	Lysine Residues in Protein			Acetylated Lysine Residues	Reference
		Total Number of Lysines	% Lysines in Total Protein	% Acetylated Lysines out of all Lysines		
**AbrB**	regulatory	11	12%	55%	48 33, 44, 73, 78 11, 50	[33] [34] this study
**DegS**	regulatory	31	8%	6%	259 223	[34] this study
**DegU**	regulatory	13	6%	15%	195 181	[34] this study
**GtaB**	biosynthetic enzyme	24	8%	29%	273, 289 186, 251 83, 186, 289 81, 83, 89, 191	[10] [33] [34] this study
**SlrR**	regulatory	15	10%	7%	48	this study
**Spo0A**	regulatory	20	7%	5%	203	this study
**TasA**	matrix assembly	34	13%	9%	216, 201 144, 152, 209	[33] this study
**YmcA**	regulatory	14	10%	21%	41, 64, 133	this study
**YmdB**	regulatory	22	8%	18%	19, 246 24, 140, 246	[34] this study

List of acetylated biofilm proteins found on our acetylome. Also listed is the percent lysine residue content in each protein, the percent of total lysine residues that were found acetylated, and the specific acetylated lysine sites. Included are acetylated lysine residues identified in other *B*. *subtilis* acetylome studies [10, 34, 38].

### The acetylated lysine residue K64 in YmcA is critical for its function in biofilm formation

**Fig 5:**
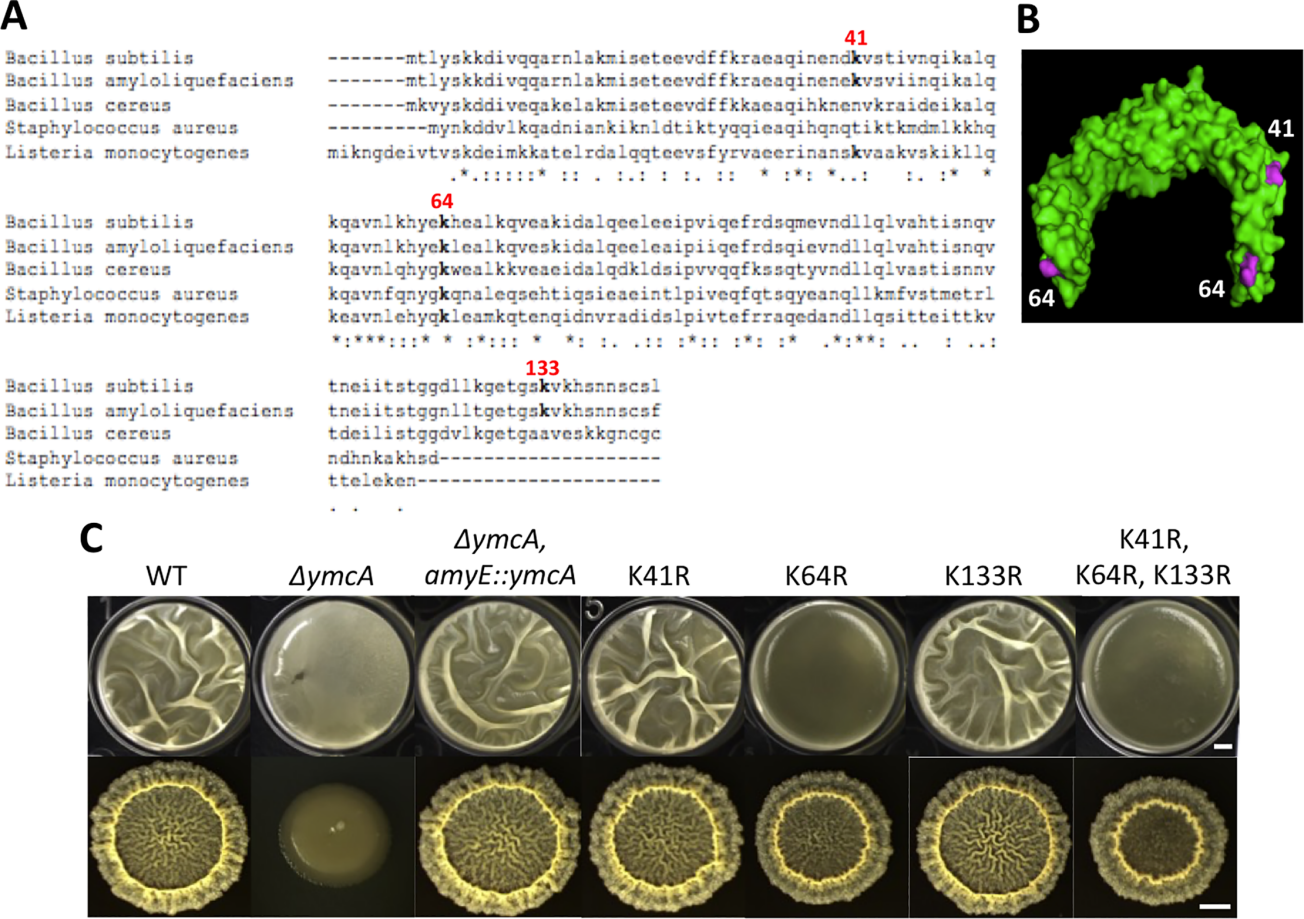
The acetylated lysine residue K64 in YmcA is important for its function in biofilm induction. **(A)** Clustal sequence alignment of YmcA homologs demonstrating conservation of sequences and acetylated lysine residues. Acetylated lysine residues are in bold and numbered. **(B)** The crystal structure of *B*. *subtilis* YmcA (PDB_2PIH) in the homodimer conformation (PyMol). Surface-exposed acetylated lysine residues (characterized in the acetylome) are highlighted in purple. K133 is not shown because the secondary structure containing K133 is missing in the crystal structure. **(C)** Comparison of biofilm formation of *B*. *subtilis* variants bearing various lysine codon substitutions (as indicated) in *ymcA*. Top, pellicle biofilm formation; Bottom, colony biofilm formation. The scale bar in the upper panel, 200 μm; the scale bars in the lower panel, 5 mm. The *ymcA*(K64R) variant and the triple mutant (K41R/K64/K133) showed a strong biofilmm defect, indicating the importance of the acetylated lysine residue K64 for YmcA funcationality.

From the above results, we speculated that certain proteins important for biofilm formation must be acetylated and/or deacetylated as a regulatory mechanism for their activities. Interestingly, in our acetylome analyses, some biofilm proteins demonstrated a significant reduction in the acetylation level in both the Δ*acuA* and Δ*pta* deletion mutants (Table 4). To further test our hypothesis, we took a targeted approach by investigating two individual acetylated proteins, GtaB and YmcA, in part because they had the most significant reduction in acetylation and are already known to be essential for biofilm formation (Table 4). It was also because GtaB and YmcA are believed to play important but distinct roles in *B*. *subtilis* biofilm formation. YmcA (also named as RicA [52]) is a member of a three-protein complex (YmcA-YlbF-YaaT) shown to be involved in a variety of biological processes including competence, sporulation, carbon metabolism, and biofilm formation in *B*. *subtilis* [52–54]. In previous studies, the complex has been shown to play a key regulatory role in biofilm formation by interacting with an endoribonuclease RNaseY, which globally regulates mRNA stability including that of *sinR* [54], In a parallel study, another mechanism was proposed in that the YmcA protein complex interferes with the phosphorelay, which controls *B*. *subtilis* cell development [53]. YmcA is highly conserved in various Gram-positive bacteria (Fig 5A). In addition to our acetylome analysis, the YmcA homolog was also found to be acetylated in the closely related *B*. *amyloliquefaciens* [38].

**Table 4:** Relative ratios in the acetylation level of biofilm and motility proteins comparing wild type and the acetylation mutants Δ*pta* and Δ*acuA*.

Biofilm Protein	*Δpta* acetylation ratio	*ΔacuA* acetylation ratio	Motility Protein	*Δpta* acetylation ratio	*ΔacuA* acetylation ratio
**AbrB**	1.000	0.988	**DegS**	1.004	1.107
**DegS**	1.004	1.107	**DegU**	1.085	0.991
**DegU**	1.085	0.991	**FliD**	No ratio	No ratio
**GtaB**	0.816	0.597	**FliF**	No ratio	No ratio
**SlrR**	No ratio	No ratio	**FliG**	1.488	1.175
**Spo0A**	No ratio	No ratio	**FliH**	No ratio	No ratio
**TasA**	No ratio	No ratio	**FliJ**	0.899	0.980
**YmcA**	0.717	0.977	**FlgG**	1.393	1.358
**YmdB**	1.016	1.009	**Hag**	1.545	1.555
			**SwrAA**	No ratio	No ratio
			**SwrC**	1.211	1.131
**AVERAGE**	**0.940**	**0.945**	**AVERAGE**	**1.232**	**1.185**

Calculated ratios in the acetylation levels of biofilm and motility proteins in Δ*pta* and Δ*acuA* mutants compared to the wild type. A ratio above 1.0 indicates an increase in acetylation of the protein compared to the wild type, while a ratio below 1.0 indicates a decrease in acetylation. Each ratio is the average ratio from all identified acetylated peptides of that particular protein. Some proteins did not show a ratio likely due to that the levels of acetyl modification are lower than the detection limit.

To test if any of the three acetylated lysine residues are important for YmcA activity in biofilm regulation, we performed site-directed mutagenesis on those lysine residues (Fig 5A). Each of the three acetylated lysine residues (K41, K64, and K133) was mutated to arginine, a structurally similar and positively charged residue. This substitution is predicted to abolish the ability of the residue to be acetylated while retaining similar biochemical properties. The *ymcA* mutant allele was integrated at the *amyE* locus on the chromosome of Δ*ymcA* for complementation. Those complemented strains were then used to test biofilm robustness. Interestingly, the K64R mutant showed a biofilm defect in both pellicle and colony biofilm formation, though the defect was stronger in the pellicle biofilm than in the colony biofilm (Fig 5C). The other two point mutants (K41R and K133R) did not show any noticeable biofilm defect while the triple mutant (K41R/K64R/K133R) was very similar to the K64R mutant (Fig 5C). Similar biofilm phenotypes were also seen in MSgg, another commonly used biofilm-inducing minimal medium [19], indicating that the phenotypes were not medium-specific (S3 Fig).

The crystal structure of *B*. *subtilis* YmcA has been published (PDB_2PIH). It shows that the protein forms a homodimer in a horseshoe-like conformation (Fig 5B). Interestingly, at least two of three acetylated lysine residues (K41 and K64) are positioned on the surface of the protein readily accessible for acetyl group modification (Fig 5B). Further, our sequence alignment showed that the acetylated lysine residue K64, whose substitution to arginine resulted in a severe biofilm defect, is highly conserved in the YmcA homologs across multiple species (Fig 5A). Together, these data indicate that the acetylated lysine residue K64 is important for YmcA function in biofilm formation, although we do not have direct evidence yet to allow us to distinguish whether the lysine residue or the acetyl modification of the lysine residue is important for YmcA function. Further *in vitro* biochemical studies are needed to provide the direct answer to the above question.

### The acetylated lysine residue K191 in GtaB is critical for its function

**Fig 6:**
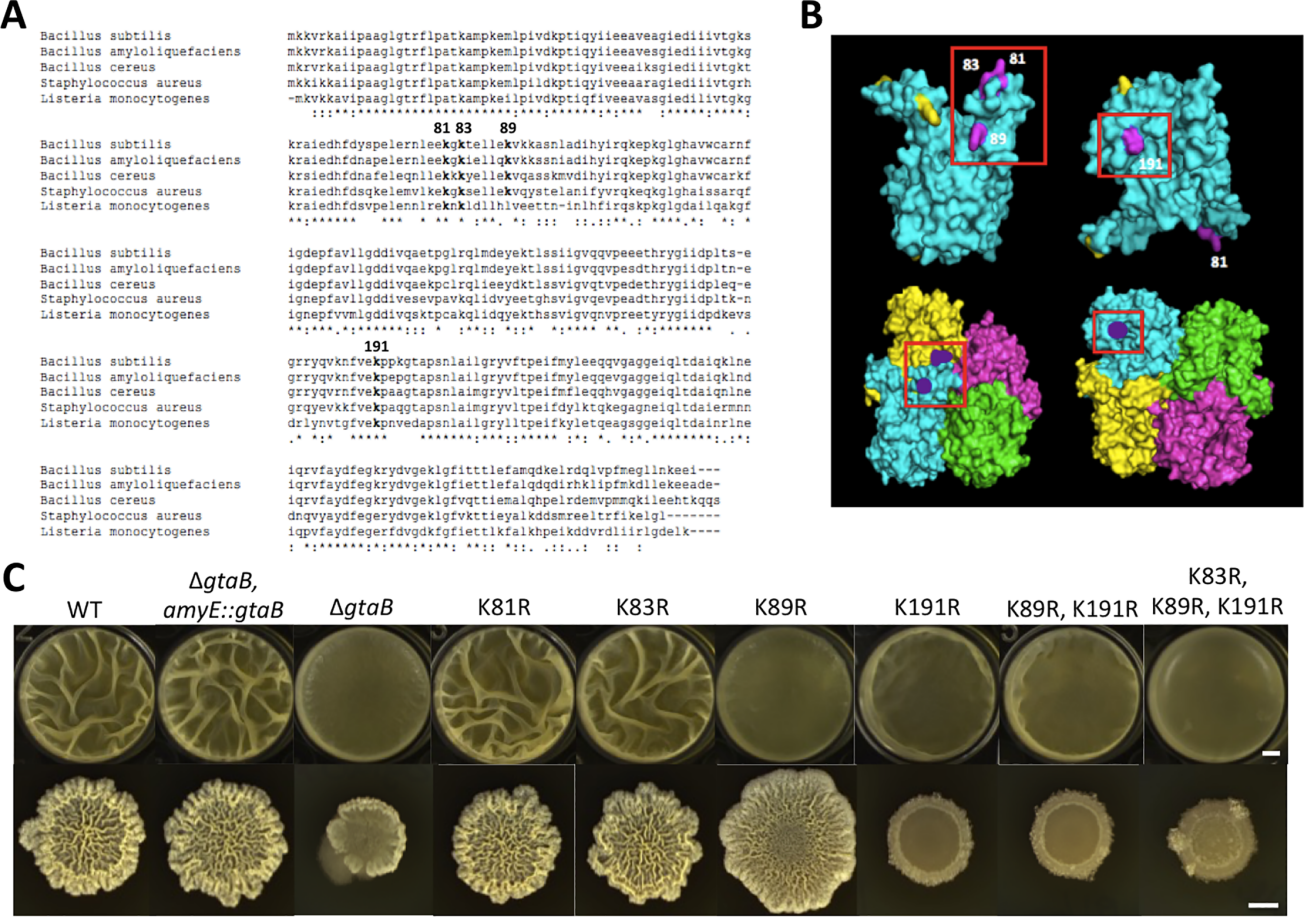
The acetylated lysine residues K89 and K191 are important for the function of GtaB in biofilm formation. **(A)** Clustal sequence alignment of GtaB homologs demonstrating conservation of sequences and acetylated lysine residues. Acetylated lysine residues are in bold and numbers indicate positions. **(A)** The crystal structure of the GtaB protein homolog in *E*. *coli* (PDB_2E3D) (PyMol). Top row is the monomer viewed from both sides flipped horizontally. Bottom row is the tetramer viewed from both sides flipped horizontally. Known acetylated lysine residues from our acetylome are highlighted in purple. Lysine residues highlighted in yellow are those identified in other acetylomes [10, 34]. **(C)** Comparison of biofilm formation of GtaB lysine residue substitution variants (as shown). Top, pellicle biofilm formation after 48 hours of incubation in LBGM; Bottom, colony biofilm formation after 72 hours of incubation on LBGM agar plates. The scale bar in the upper panel, 200 μm; the scale bars in the lower panel, 5 mm.

Another acetylated protein of interest is GtaB. GtaB is an UTP-glucose-1-phosphate uridylyltransferase that converts glucose-1-phosphate to UDP-glucose, a nucleotide sugar precursor essential for exopolysaccharide biosynthesis [55]. Our acetylome analyses showed that this protein was acetylated at multiple lysine residues: K81, K83, K89, and K191. These lysine residues are also highly conserved among GtaB homologs from different species (Fig 6A). GtaB also had the greatest reduction in the acetylation level in both the Δ*acuA* and Δ*pta* deletion mutants when compared to the wild type (Table 4). The crystal structure of a GtaB homolog protein in *E*. *coli* (PDB_2E3D[56]) is available. Its crystal structure shows that those acetylated lysine residues are surface-exposed and easily accessible to modification (Fig 6B).

To investigate the potential importance of acetyl modification of those lysine residues on GtaB function, we similarly performed site-directed mutagenesis on the lysine residues by substituting them with arginine. Strains carrying those mutant alleles were assayed for their abilities to form biofilms. Our results showed that one of the mutant alleles had a strong biofilm defect (K191R, Fig 6C). In addition, the single mutant K89R also showed a biofilm defect, albeit more severe in the pellicle than in the colony biofilm (Fig 6C). From the crystal structure of the GtaB homolog, K89 is located close to the intersection of the tetramer. We suspect that the residue K89 may have a role in tetramer formation while K191 may directly impact the enzymatic activity since it is next to the catalytic center (Fig 6B). Lastly, the K89R/K191R double and the K83R/K89R/K191R triple mutants also showed a similar strong biofilm defect (Fig 6C). Similar biofilm phenotypes were also seen in MSgg, indicating that the phenotypes were not medium-specific (S4 Fig). These results suggest that the lysine residue 191 is critical for the function of GtaB. Again, additional biochemical studies are needed in the future to characterize the regulatory role of acetyl modification of these lysine residues in GtaB.

## Discussion

In this study, we investigated the role of one of the post-translational modification mechanisms, protein lysine acetylation, in *B*. *subtilis* multicellular development. We analyzed global acetylomes of wild type *B*. *subtilis* and two global acetylation mutants grown under biofilm-inducing conditions. We characterized the motifs and diverse categories of proteins modified by lysine acetylation, in particular, a number of acetylated proteins known to be involved in swarming motility and biofilm formation. We investigated the impact of global lysine acetylation on the two multicellular processes by constructing mutants altered in one of the two acetylation mechanisms, chemical or enzymatic acetylation pathways. Many of those mutants showed a clear defect in swarming motility while some also showed a defect in biofilm formation (Figs 3 and 4). In the second half of this study, we picked two individual proteins, YmcA and GtaB, shown to be acetylated in our acetylome analysis and whose acetylation levels significantly decreased in both the Δ*pta* and Δ*acuA* global acetylation mutants. We conducted further investigations on the role of these acetylated lysine residues in their protein function. The regulatory protein YmcA was previously shown to be essential for biofilm formation [51, 54] while the UTP-glucose-1-phosphate uridylyltransferase GtaB was first shown to be essential for biofilm formation in *B*. *subtilis*. By application of site-directed mutagenesis of selected acetylated lysine residues, we demonstrated that some of these modified lysine residues are indeed important for the protein function. Collectively, our results support the idea that protein lysine acetylation plays a global role in bacterial multicellularity in *B*. *subtilis*.

### Protein lysine acetylation pathways in *B*. *subtilis* are complex and likely redundant

In addition to generating an acetylome from the wild type cells under biofilm-inducing conditions, we also generated acetylomes from two deletion mutants expected to be impaired in acetylation, Δ*acuA* and Δ*pta*. Surprisingly, we did not observe significant changes in the number of acetylated proteins between the wild type and the two acetylation mutants. We can think of a few possible explanations for this finding. First, the single deletion mutant is likely only impaired in one of the two acetylation mechanisms, either chemical or enzymatic (Fig 1). It is possible that one mechanism may compensate for the loss of the other, and *vice versa*. Second, in the case of Δ*pta*, the gene deletion could cause a build-up of acetyl-CoA, which is a substrate for enzymatic acetylation, and thus increases the rate of enzymatic acetylation. Third, AcuA is the only *B*. *subtilis* lysine acetyltransferase characterized so far [40]. But due to limited numbers of published studies on lysine acetylation mechanisms in *B*. *subtilis*, and given the presence of multiple lysine acetyltransferases in other bacterial species and eukaryotes, it is possibly that other uncharacterized lysine acetyltransferases are also involved in protein lysine acetylation in *B*. *subtilis*, which may explain why the deletion of *acuA* does not cause a significant drop in global acetylation levels.

### The putative role of protein lysine acetylation in swarming motility

Our finding that global lysine acetylation plays a role in swarming motility in *B*. *subtilis* is interesting and novel (Table 2 and Fig 3). Global lysine acetylation could impact swarming motility in a variety of ways as seen by the list of motility-related proteins found in the acetylomes in this study (Table 2). For example, DegU and DegS are two regulatory proteins found to be acetylated in our acetylome as well as in a previously published *B*. *subtilis* global acetylome [34]. The DegS-DegU two-component system regulates more than one hundred genes involved in motility and biofilm formation [57]. It regulates the expression of the *fli*, *flg*, and *che* genes for motility and chemotaxis [58]. The activity of DegS-DegU is known to be regulated by protein phosphorylation [59, 60]. Based on the characterization in this study, it is possible that acetylation is also important in regulating the activity of these two proteins. A second possibility of how lysine acetylation may regulate swarming motility is on structural proteins such as the flagellar motor proteins. In the flagellar machinery, over 25 proteins are complexed to form a motor [49]. Six of those motor proteins were found to be acetylated in our acetylome (Table 2). This could suggest that the flagellar machinery is acetylated during biofilm formation, which then alters the function of those proteins and thus blocks motility to facilitate the transition to the sessile state. It is interesting and also intriguing to point out that most of the flagellar machinery proteins were not found on the acetylome generated under standard growth conditions in a previous study [34].

### Lysine acetylation could impact two different aspects of biofilm development: Gene regulation and polysaccharide biosynthesis

Multiple proteins involved in biofilm formation were found to be acetylated. We picked two individual proteins, YmcA and GtaB, from our acetylome and showed that substitution of the selected acetylated lysine residues with arginine resulted in the mutant alleles that demonstrated strong biofilm defects. YmcA and GtaB represent two important, but distinct aspects of biofilm development in *B*. *subtilis*: gene regulation and exopolysaccharide biosynthesis. YmcA is known to indirectly regulate the expression of the key biofilm matrix genes while GtaB is an enzyme essential for biosynthesis of UDP-glucose, a critical nucleotide sugar precursor for exopolysaccharide biosynthesis [54, 55, 61]. YmcA binds to and forms a complex with two other proteins, YlbF and YaaT [52–54], and the complex functions in a variety of processes including biofilm formation, sporulation, and competence [51, 53, 54, 62]. It is possible that acetyl modification of YmcA influences its binding with the two other proteins. Similarly, acetylation may regulate the activity of GtaB by enabling conformational changes, tetramer formation, and/or exposure of the catalytic site. Lastly, it is important to acknowledge that substitutions of the acetylated lysine residues and the observed phenotypes from the subsequent mutant alleles show the importance of those lysine residues in protein activity, but do not directly imply that acetyl modification of the lysine residues is a regulatory mechanism for the protein function. Although this is an accepted practice in several previous studies, further biochemical and *in vitro* studies are needed to directly address how lysine acetylation impacts the activity of those proteins. Recent studies have begun to look at acetylation stoichiometry and saturation, which may provide a better picture of *in vivo* acetylation dynamics at the global, protein-specific, and residue-specific levels. Improving modification methods, such as incorporating click chemistry for quicker and more accurate screens of protein lysine acetylation will provide more insight into how this modification mechanism regulates the activity of proteins.

### Protein lysine acetylation mechanisms link cell metabolism to multicellular behaviors

Protein lysine acetylation is closely linked to central metabolism in that the enzymatic acetylation uses acetyl-CoA, the end product of glycolysis, as an acetyl group donor while chemical acetylation uses acetyl-phosphate, the intermediate metabolite in the carbon-overflow pathway, as a donor of the acetyl group [47]. Previously published studies have shown that lysine acetylation targets proteins and enzymes involved in cellular metabolism [3, 6, 33]. These metabolic processes include glycolysis, the citric acid cycle, biosynthetic pathways for amino acids and nucleotides, etc. One previous study also suggested that the bacterium *B*. *subtilis* may use lysine acetylation as a regulatory mechanism for the cytoskeleton protein MreB, linking cellular metabolism to cell size regulation [34].

In this study, we provided genetic evidence for the putative novel effect of lysine acetyl modification on proteins involved in swarming and biofilm formation, and suggested a link between protein lysine acetylation, acetyl-CoA homeostasis, and bacterial multicellular behaviors. Like other types of post-translational modification mechanisms, acetylation is an energy-conservative mechanism that allows for quick response to environmental changes. However, distinct from other post-translational modifications, acetylation is more closely linked to, and thus reflects, the internal metabolic status of the cells and overall nutrient status of the environment.

## Supporting information

S1 Fig Distribution of the number of acetylated lysine residues in all acetylated proteins characterized in this study. *x*-axis represents the number of acetylated lysine residues in each candidate protein. *y*-axis represents the total number of proteins showing the designated number of acetylated lysine residues.
(PDF)

S2 Fig Deletion of *pta* or *ackA* for the chemical acetylation enzymes had a growth defect. Growth curve of various acetylation mutants grown in shaking LB broth at 37°C. OD_600_ values of the cultures were measured every 15 minutes over a period of 15 hours. Results are the mean of 8 samples with bars representing calculated standard deviations. Chemical acetylation mutants Δ*pta*, Δ*pta*Δ*acuA*, and Δ*ackA*, showed mild to modest growth defects compared to wild type while the growth of the enzymatic acetylation mutants Δ*acuA*, Δ*acuC*, Δ*srtN*, and Δ*acuC*Δ*srtN* is comparable to the wild type.
(PDF)

S3 Fig Acetylated lysine residues in YmcA are important for biofilm formation in MSgg. Comparison of biofilm formation of YmcA lysine residue mutants in MSgg. Top, colony biofilm formation. Bottom, pellicle biofilm formation. Defective biofilm phenotypes were observed in the YmcA(K64R) and YmcA triple lysine mutants indicating the importance of the acetylated K64 residue for the function of YmcA in biofilm formation.
(PDF)

S4 Fig Acetylated lysine residues in GtaB are important for biofilm formation in MSgg. Comparison of biofilm formation of GtaB lysine residue mutants in MSgg. Top, colony biofilm formation. Bottom, pellicle biofilm formation. Clear biofilm phenotypes were observed in the GtaB(K89R), GtaB(K191R), GtaB double and triple mutants indicating the importance of the acetylated K89 and K191 residues for the function of GtaB in biofilm formation.
(PDF)

S1 Table Raw acetylome data. (PDF)
