# Supplementary material for: Protein lysine acetylation plays a regulatory role in Bacillus subtilis multicellularity
Source: PLoS One. 2018 Sep 28;13(9):e0204687. doi: 10.1371/journal.pone.0204687 (PMC6161898; doi:10.1371/journal.pone.0204687)
Supplement: S1 Fig — x-axis represents the number of acetylated lysine residues in each candidate protein. y-axis represents the total number of proteins showing the designated number of acetylated lysine residues. (PDF) [file pone.0204687.s001.pdf]

Supplemental Figure Legends

Supplement Figure 1. Distribution of the number of acetylated lysine residues in all acetylated proteins characterized in this study. *x*-axis represents the number of acetylated lysine residues in each candidate protein. *y*-axis represents the total number of proteins showing the designated number of acetylated lysine residues.

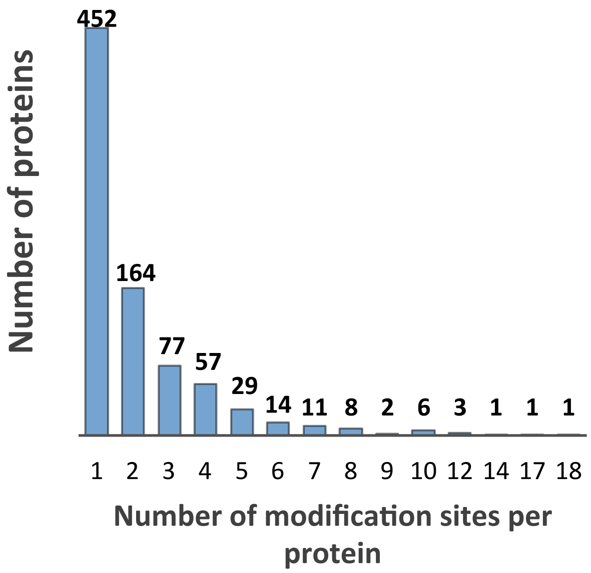

Supplement Figure 1
