# Supplementary material for: Protein lysine acetylation plays a regulatory role in Bacillus subtilis multicellularity
Source: PLoS One. 2018 Sep 28;13(9):e0204687. doi: 10.1371/journal.pone.0204687 (PMC6161898; doi:10.1371/journal.pone.0204687)
Supplement: S2 Fig — Growth curve of various acetylation mutants grown in shaking LB broth at 37°C. OD600 values of the cultures were measured every 15 minutes over a period of 15 hours. Results are the mean of 8 samples with bars representing calculated standard deviations. Chemical acetylation mutants Δpta, ΔptaΔacuA, and ΔackA, showed mild to modest growth defects compared to wild type while the growth of the enzymatic acetylation mutants ΔacuA, ΔacuC, ΔsrtN, and ΔacuCΔsrtN is comparable to the wild type. (PDF) [file pone.0204687.s002.pdf]

Supplement Figure 2. Deletion *pta* and *ackA* for the chemical acetylation enzymes had a growth defect. Growth curved of various acetylation mutants grown in shaking LB broth at 37°C. OD_{600} values of the cultures were measured every 15 minutes over a period of 15 hours. Results are the mean of 8 samples with bars representing calculated standard deviations. Chemical acetylation mutants Δ*pta,* Δ*pta*Δ*acuA,* and Δ*ack,* showed mild to modest growth defects compared to wild type while the growth of the enzymatic acetylation mutants Δ*acuA,* Δ*acuC,* Δ*srtN,* and Δ*acuC*Δ*srtN* is comparable to the wild type.

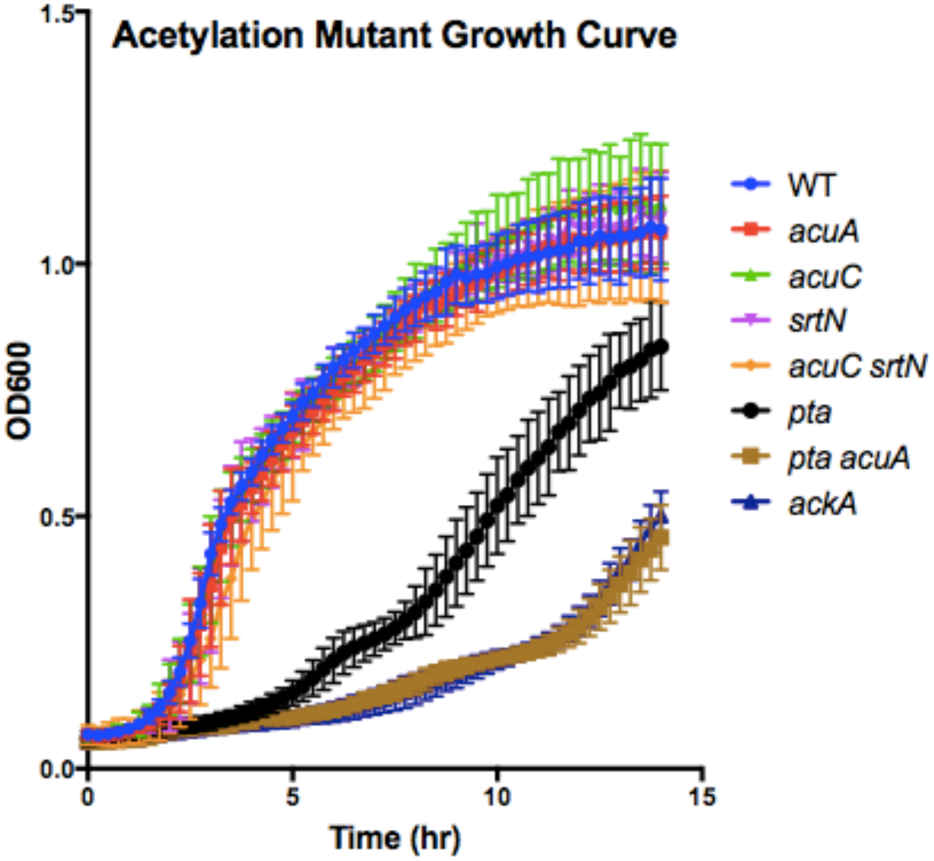

Supplement Figure 2
