# Supplementary material for: Protein lysine acetylation plays a regulatory role in Bacillus subtilis multicellularity
Source: PLoS One. 2018 Sep 28;13(9):e0204687. doi: 10.1371/journal.pone.0204687 (PMC6161898; doi:10.1371/journal.pone.0204687)
Supplement: S3 Fig — Comparison of biofilm formation of YmcA lysine residue mutants in MSgg. Top, colony biofilm formation. Bottom, pellicle biofilm formation. Defective biofilm phenotypes were observed in the YmcA(K64R) and YmcA triple lysine mutants indicating the importance of the acetylated K64 residue for the function of YmcA in biofilm formation. (PDF) [file pone.0204687.s003.pdf]

Supplement Figure 3. Acetylated lysine residues in YmcA is important for biofilm formation in MSgg. Comparison of biofilm formation of YmcA lysine residue mutants in MSgg minimal media. Top, colony biofilm formation. Bottom, pellicle biofilm formation. Defective biofilm phenotypes were observed in the YmcA(K64R) and YmcA triple lysine mutants indicating the importance of the acetylated K64 residue for the function of YmcA in biofilm formation.

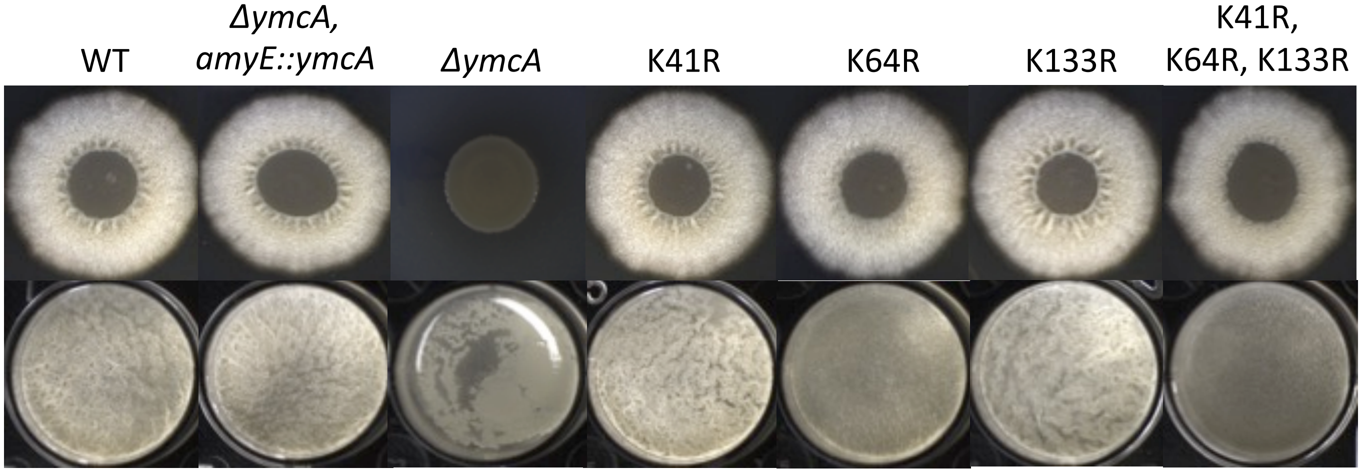

Supplement Figure 3
