# Supplementary material for: Protein lysine acetylation plays a regulatory role in Bacillus subtilis multicellularity
Source: PLoS One. 2018 Sep 28;13(9):e0204687. doi: 10.1371/journal.pone.0204687 (PMC6161898; doi:10.1371/journal.pone.0204687)
Supplement: S4 Fig — Comparison of biofilm formation of GtaB lysine residue mutants in MSgg. Top, colony biofilm formation. Bottom, pellicle biofilm formation. Clear biofilm phenotypes were observed in the GtaB(K89R), GtaB(K191R), GtaB double and triple mutants indicating the importance of the acetylated K89 and K191 residues for the function of GtaB in biofilm formation. (PDF) [file pone.0204687.s004.pdf]

Supplement Figure 4. Acetylated lysine residues in GtaB is important for biofilm formation in MSgg. Comparison of biofilm formation of GtaB lysine residue mutants in MSgg minimal media. Top, colony biofilm formation. Bottom, pellicle biofilm formation. Clear biofilm phenotypes were observed in the GtaB(K89R), GtaB(K191R), GtaB double and triple mutants indicating the importance of the acetylated K89 and K191 for the function of GtaB in biofilm formation.

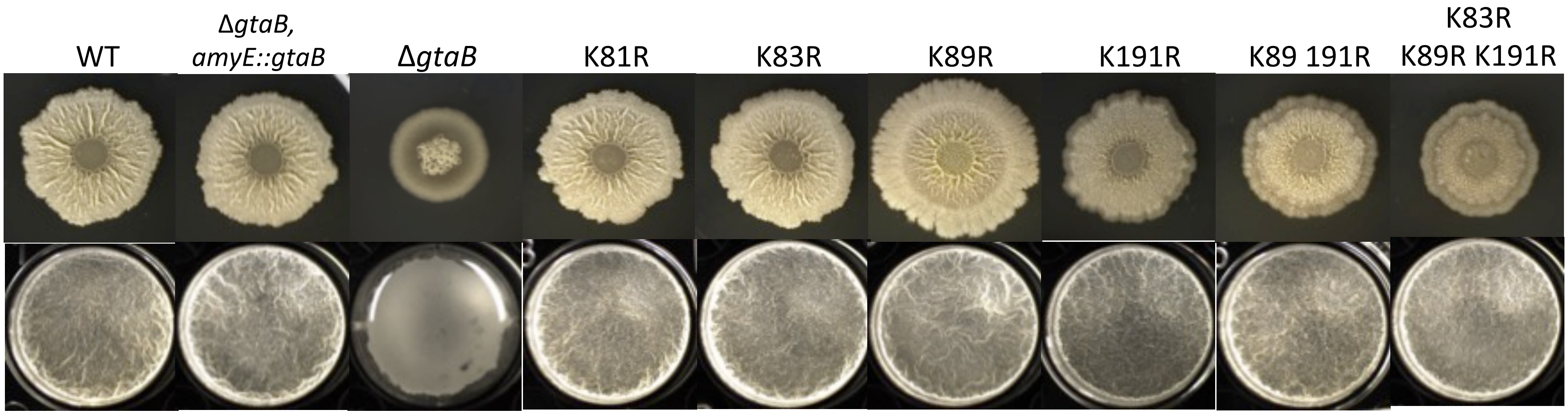

Supplement Figure 4
