# Supplementary material for: Protein lysine acetylation plays a regulatory role in Bacillus subtilis multicellularity
Source: PLoS One. 2018 Sep 28;13(9):e0204687. doi: 10.1371/journal.pone.0204687 (PMC6161898; doi:10.1371/journal.pone.0204687)
Supplement: S1 Table — (PDF) [file pone.0204687.s005.pdf]

Protein	*pta* relative intensity	*acuA* relative intensity	S3610-1 relative intensity	S3610-2 relative intensity	*pta*/S3610-1 Ratio	*acuA*/S3610-1 Ratio
yezG	0.814	0.744	1.404	1.039	0.580	0.530
mprF	0.955	1.218	1.384	0.443	0.690	0.880
sftA	0.934	0.993	0.983	1.091	0.950	1.010
accD	0.977	0.864	1.028	1.131	0.950	0.840
ycnJ	1.070	0.961	0.781	1.188	1.370	1.230
ytcI	1.001	1.032	0.965	1.002	1.040	1.073
yhaQ	0.888	1.092	1.020	1.000	0.870	1.070
yjhB	0.828	0.839	1.090	1.243	0.760	0.770
yugG	1.178	0.959	0.913	0.950	1.290	1.050
maeN	0.922	0.960	0.941	1.176	0.980	1.020
guaC	1.310	0.972	0.825	0.893	1.640	1.225
ydhE	0.773	1.220	1.085	0.922	0.715	1.135
ydiF	0.845	0.947	0.928	1.281	0.910	1.020
yraA	0.693	0.896	1.067	1.344	0.650	0.840
yfmQ	1.008	0.996	0.966	1.030	1.043	1.033
yfmS	0.989	1.102	0.755	1.155	1.310	1.460
yfmT	1.050	0.975	0.839	1.135	1.275	1.225
yetN	0.819	0.843	1.187	1.151	0.690	0.710
gatA	0.892	0.892	0.883	1.333	1.010	1.010
gatC	0.975	1.005	0.995	1.025	0.980	1.010
sbcC	0.928	1.045	1.111	0.917	0.845	0.945
yisK	0.722	1.078	1.078	1.121	0.670	1.000
yitH	0.901	1.013	1.013	1.073	0.890	1.000
yvcA	1.014	0.963	1.021	1.002	0.995	0.945
lutB	0.967	1.091	0.948	0.994	1.020	1.150
hit	0.857	1.061	1.020	1.061	0.840	1.040
yhaM	0.995	0.995	0.939	1.070	1.060	1.060
yheA	0.877	0.855	1.096	1.173	0.800	0.780
yhdW	0.925	0.978	1.075	1.022	0.860	0.910
yhfE	0.912	1.040	1.061	0.987	0.860	0.980
lplJ	0.794	1.116	0.946	1.144	0.840	1.180
phoE	0.672	1.081	1.050	1.197	0.640	1.030
typA	0.989	1.058	0.936	1.017	1.057	1.127
ylaL	0.927	0.979	1.042	1.052	0.890	0.940
ylaN	0.851	1.019	0.842	1.288	1.010	1.210
azlB	1.051	1.083	1.072	0.794	0.980	1.010
gatB	0.924	0.916	1.023	1.137	0.913	0.902
ybyB	0.578	0.707	0.584	2.131	0.990	1.210
ybfF	1.077	1.244	0.792	0.887	1.360	1.570
yfbQ	0.892	0.787	1.001	1.319	0.890	0.790
ycgT	0.863	0.965	1.127	1.044	0.770	0.860

swrC	1.100	1.029	0.995	0.875	1.113	1.050
yeeF	0.944	1.108	1.026	0.923	0.920	1.080
yeeI	1.016	0.901	1.047	1.037	0.970	0.860
yfjO	0.905	0.958	1.053	1.084	0.860	0.910
yfhC	0.905	0.914	1.045	1.136	0.868	0.878
yjbC	0.542	0.578	0.722	2.159	0.750	0.800
yjbG	0.876	0.984	1.019	1.120	0.860	0.965
ppnKA	0.934	1.040	1.061	0.966	0.880	0.980
yjcN	1.093	0.919	0.792	1.196	1.380	1.160
yjcS	0.677	0.979	0.657	1.688	1.030	1.490
yjdG	1.222	1.171	0.855	0.752	1.430	1.370
htpX	0.982	1.064	0.909	1.045	1.080	1.170
mtnB	0.870	1.183	0.978	0.968	0.890	1.210
moaE	0.936	0.933	1.117	1.013	0.840	0.830
yknX	1.153	1.022	0.762	1.063	1.540	1.370
ykpA	1.037	1.007	0.988	0.968	1.050	1.020
ykzG	1.076	0.928	0.868	1.128	1.240	1.070
rplS	1.146	0.948	0.939	0.967	1.220	1.010
rbgA	1.150	0.891	1.173	0.786	0.980	0.760
pyrH	1.024	0.839	0.976	1.161	1.050	0.860
dxr	0.904	1.101	0.941	1.054	0.960	1.170
rasP	0.967	0.925	0.833	1.275	1.160	1.110
proS	1.101	1.104	0.925	0.870	1.213	1.223
rnjB	1.175	0.995	0.980	0.850	1.243	1.047
ymfH	0.902	0.938	0.893	1.268	1.010	1.050
ymfK	0.925	1.076	0.740	1.259	1.250	1.455
rny	1.085	0.842	1.044	1.028	1.040	0.810
ymdB	0.990	0.995	0.973	1.042	1.075	1.060
miaB	1.014	1.122	0.905	0.959	1.120	1.240
ymcA	0.764	1.041	0.960	1.235	0.800	1.085
pksS	0.633	0.869	1.252	1.246	0.505	0.690
yoaF	1.245	1.172	0.915	0.668	1.360	1.280
yozO	1.048	1.150	1.018	0.784	1.030	1.130
yomN	0.712	1.394	0.962	0.933	0.740	1.450
sunS	0.915	1.139	1.072	0.874	0.855	1.065
yokI	0.961	1.220	1.034	0.786	0.930	1.180
yokD	0.791	0.891	1.114	1.203	0.710	0.800
yqzD	1.126	0.958	1.161	0.755	0.970	0.825
yqzC	1.130	0.920	1.000	0.950	1.130	0.920
mtnN	1.262	1.036	1.026	0.677	1.230	1.010
yrrT	0.775	1.318	0.891	1.016	0.870	1.480
aspS	0.995	0.932	0.905	1.167	1.100	1.030
hisS	0.966	0.984	0.878	1.172	1.100	1.120
yrvJ	0.935	0.880	1.087	1.098	0.860	0.810
secDF	0.940	1.120	0.846	1.095	1.117	1.347

yrbF	1.064	0.840	1.120	0.975	0.950	0.750
yuaE	0.762	0.798	0.907	1.533	0.840	0.880
pncB	0.916	1.022	0.881	1.181	1.040	1.160
yueH	0.786	1.091	0.983	1.140	0.800	1.110
yueB	1.103	0.887	0.987	1.023	1.126	0.912
besA	0.926	1.143	1.143	0.789	0.810	1.000
yutI	0.797	0.888	0.830	1.485	0.960	1.070
lipA	0.996	1.036	1.105	0.863	0.905	0.985
yurK	1.015	1.116	0.846	1.023	1.200	1.320
yurQ	0.877	0.917	0.791	1.415	1.110	1.160
sufB	0.939	1.025	1.040	0.997	0.900	0.985
sufD	1.046	0.993	0.871	1.089	1.200	1.140
yusI	0.705	0.784	1.136	1.375	0.620	0.690
fadE	0.710	2.179	0.351	0.759	2.020	6.200
fadA	0.861	2.206	0.606	0.327	1.420	3.640
cssR	0.809	1.009	0.909	1.273	0.890	1.110
yvgN	0.690	0.976	1.190	1.143	0.580	0.820
cadA	0.856	1.338	0.719	1.086	1.190	1.860
iolW	0.478	0.539	0.870	2.113	0.550	0.620
yvbH	0.957	0.809	1.064	1.170	0.900	0.760
yvbJ	0.806	0.797	1.067	1.330	0.800	0.785
lutC	0.952	1.227	0.915	0.906	1.040	1.340
yvcT	0.704	0.943	1.194	1.158	0.590	0.790
ytoQ	0.895	1.032	1.053	1.021	0.850	0.980
yjoA	0.609	0.954	1.097	1.339	0.565	0.875
fabF	0.868	1.063	0.976	1.093	0.890	1.090
yfmC	1.240	0.989	0.758	1.013	1.635	1.305
ytpP	0.386	0.772	1.286	1.556	0.300	0.600
htrA	1.117	0.933	1.227	0.724	0.910	0.760
ytmB	0.927	1.150	1.173	0.751	0.790	0.980
yjjA	0.869	1.226	1.190	0.714	0.730	1.030
gapB	0.819	0.945	1.050	1.186	0.780	0.900
mgtE	0.876	0.735	1.081	1.308	0.810	0.680
apt	0.970	0.970	0.844	1.215	1.150	1.150
ykaA	1.149	1.052	1.005	0.793	1.143	1.050
yeeB	1.031	1.017	1.356	0.597	0.760	0.750
yoeA	0.944	1.093	1.061	0.902	0.890	1.030
yfkO	0.925	0.952	0.680	1.442	1.360	1.400
ytkP	0.839	0.955	0.893	1.313	0.940	1.070
hprK	1.051	1.051	1.194	0.704	0.880	0.880
mapB	1.029	1.019	0.962	0.990	1.070	1.060
pgl	0.815	0.979	1.055	1.151	0.770	0.925
prkC	0.937	1.111	0.966	0.986	0.970	1.150
nagP	1.210	1.201	0.864	0.726	1.400	1.390
alaS	1.022	0.955	1.053	0.971	0.982	0.918

cymR	0.824	1.049	0.980	1.147	0.840	1.070
pfkA	1.082	0.876	1.131	0.911	0.970	0.815
ycdA	1.225	0.917	1.281	0.577	0.958	0.718
yjqA	1.047	0.811	1.161	0.982	0.910	0.710
cccB	0.877	0.930	1.070	1.123	0.820	0.870
ytqB	0.959	0.969	1.031	1.041	0.930	0.940
pfyP	0.984	0.984	1.047	0.984	0.940	0.940
yvlB	0.960	1.040	0.889	1.111	1.080	1.170
yerH	1.067	1.078	1.078	0.776	0.990	1.000
yjlC	0.965	1.077	0.928	1.030	1.040	1.160
dhaS	0.765	1.158	1.093	0.984	0.700	1.060
yjiA	0.729	0.862	0.701	1.709	1.040	1.230
rpmF	1.128	0.876	0.953	1.044	1.190	0.925
yloA	1.029	0.957	0.922	1.092	1.115	1.035
mltG	0.908	1.161	1.055	0.876	0.860	1.100
ohrA	0.983	1.148	0.973	0.895	1.010	1.180
sat	0.773	1.516	0.905	0.806	0.855	1.675
bdhA	0.929	1.154	0.848	1.069	1.095	1.360
ydjI	1.312	1.023	0.905	0.760	1.450	1.130
ftsE	1.080	0.893	1.056	0.972	1.025	0.840
glmM	0.862	0.916	0.990	1.232	0.870	0.925
yceH	1.121	1.139	0.804	0.936	1.394	1.420
rok	1.040	0.974	0.937	1.049	1.110	1.040
rsbRB	1.124	1.030	0.858	0.987	1.310	1.200
ydiS	1.118	1.036	1.026	0.821	1.090	1.010
ezrA	0.924	0.893	1.005	1.177	0.930	0.897
year	1.020	0.955	1.146	0.880	0.935	0.895
nadK2	0.898	1.127	0.886	1.089	1.010	1.280
ytpR	0.962	0.914	0.952	1.171	1.010	0.960
ytsJ	0.847	0.944	1.070	1.139	0.797	0.883
yfjR	0.906	0.979	1.208	0.906	0.750	0.810
dapH	1.120	1.236	1.047	0.597	1.070	1.180
purU	1.082	0.948	1.031	0.938	1.050	0.920
polA	0.935	0.921	1.014	1.130	0.933	0.920
yvrP	1.022	1.065	0.866	1.048	1.180	1.230
ytkD	0.957	0.935	1.087	1.022	0.880	0.860
mnmA	1.008	0.986	1.031	0.976	0.980	0.960
azoR1	1.020	1.329	0.718	0.934	1.420	1.850
coaBC	0.918	1.027	0.909	1.145	1.010	1.130
yfkC	0.903	0.745	1.050	1.302	0.860	0.710
rplL	1.164	1.039	1.033	0.764	1.128	1.010
hag	1.185	1.193	0.722	0.900	1.644	1.652
rpsK	0.983	1.096	1.062	0.859	0.935	1.030
gyrB	1.019	1.019	0.934	1.029	1.093	1.090
gyrA	1.176	0.986	0.828	1.010	1.420	1.190

pyrB	1.589	0.693	1.067	0.651	1.490	0.650
rpmA	1.079	0.920	0.861	1.141	1.255	1.065
sigA	0.731	0.731	1.434	1.104	0.510	0.510
dnaI	0.876	1.085	0.995	1.045	0.880	1.090
spo0F	0.627	0.951	0.980	1.441	0.640	0.970
fumC	0.992	0.939	0.955	1.114	1.052	0.992
cotC	0.429	0.435	0.621	2.516	0.690	0.700
sdhA	1.148	1.102	0.839	0.911	1.375	1.310
nadE	0.897	1.084	0.935	1.084	0.960	1.160
dacA	1.086	0.841	1.004	1.068	1.085	0.835
hupA	0.831	1.125	0.943	1.100	0.883	1.193
ptsI	0.871	0.950	0.996	1.182	0.880	0.964
abrB	0.989	0.977	0.903	1.132	1.100	1.090
gapA	0.847	0.908	0.903	1.342	0.937	1.003
citB	1.046	1.291	0.890	0.773	1.209	1.450
glmS	1.114	0.847	1.177	0.862	1.047	0.813
gpsB	0.926	0.897	1.132	1.045	0.843	0.793
rplX	1.242	1.050	1.083	0.625	1.357	1.113
thyA1	0.874	1.126	0.866	1.134	1.010	1.300
alr1	0.862	0.923	1.026	1.190	0.840	0.900
ogt	1.049	0.996	0.897	1.058	1.170	1.110
gntZ	0.994	0.955	0.783	1.268	1.270	1.220
purH	1.370	1.144	0.925	0.560	1.495	1.235
glnA	1.029	0.898	0.998	1.075	1.033	0.903
rpmC	1.130	1.022	1.087	0.761	1.040	0.940
rpsQ	0.989	0.928	1.392	0.691	0.720	0.665
rplN	1.143	1.101	1.181	0.575	0.980	0.935
rplE	1.088	0.955	0.943	1.015	1.158	1.013
rpsN1	0.613	0.878	1.394	1.115	0.440	0.630
rpsH	1.167	0.873	1.005	0.955	1.170	0.870
ctaA	1.003	0.945	1.153	0.899	0.870	0.820
pyrG	1.203	0.936	1.028	0.833	1.170	0.910
fbaA	0.991	1.041	1.005	0.963	0.993	1.043
polC	1.138	0.874	0.825	1.163	1.380	1.060
tagF	1.043	0.934	1.024	1.000	1.040	0.922
ldh	0.894	1.160	0.766	1.180	1.178	1.520
degS	0.970	1.069	1.102	0.860	0.880	0.970
degU	1.064	0.972	1.023	0.941	1.040	0.950
glmU	1.112	0.928	0.962	0.998	1.170	0.975
prs	0.905	0.905	1.028	1.162	0.880	0.880
rplP	1.096	1.017	1.130	0.757	0.970	0.900
trxA	0.722	1.044	1.035	1.199	0.715	1.030
grpE	1.030	1.000	1.010	0.960	1.020	0.990
odhB	1.055	1.006	1.172	0.767	0.917	0.873
adk	0.911	0.999	0.924	1.166	1.025	1.125

ribBA	0.880	1.165	0.815	1.141	1.080	1.430
dnaJ	1.400	0.851	0.706	1.044	2.080	1.220
dnaK	1.020	1.083	1.027	0.870	1.008	1.064
ftsZ	0.740	1.013	0.881	1.366	0.840	1.150
infB	1.008	0.953	0.912	1.127	1.105	1.045
argR	0.854	0.984	0.928	1.234	0.920	1.060
rsbV	0.748	0.844	0.959	1.448	0.780	0.880
rsbW	0.673	0.925	1.201	1.201	0.560	0.770
pheS	1.063	0.953	0.932	1.053	1.140	1.030
pheT	1.010	0.970	0.971	1.048	1.043	1.007
glpD	1.008	0.990	0.958	1.044	1.069	1.050
pgcA	0.805	0.923	1.067	1.204	0.755	0.868
thrS	0.936	1.171	1.040	0.853	0.900	1.125
murB	1.004	0.796	0.991	1.209	1.015	0.805
folE	1.057	0.925	0.999	1.019	1.060	0.925
hom	0.945	1.019	1.004	1.032	0.940	1.015
tal	0.908	1.125	1.231	0.736	0.753	0.927
murAB	0.977	1.178	0.913	0.932	1.070	1.290
rplO	1.120	1.026	0.855	1.000	1.310	1.200
rpmD	1.167	0.978	1.080	0.775	1.087	0.920
map	0.899	1.044	1.034	1.023	0.870	1.010
ptsG	0.675	1.015	0.619	1.690	1.090	1.640
rpmJ	0.539	0.991	1.739	0.730	0.310	0.570
rpsM	1.290	0.795	0.894	1.021	1.445	0.890
rpoA	1.219	0.961	0.826	0.993	1.552	1.210
fliJ	0.927	1.010	1.042	1.021	0.890	0.970
gltC	0.875	0.966	0.911	1.248	0.960	1.060
aroA	1.128	0.859	1.342	0.671	0.840	0.640
obg	0.924	0.948	0.803	1.325	1.150	1.180
rpsB	1.198	0.941	1.059	0.802	1.143	0.893
rpsC	1.474	0.896	0.915	0.715	1.708	1.012
rspD	1.175	1.006	1.063	0.755	1.166	1.004
rspE	1.154	0.938	0.996	0.913	1.173	0.950
rpsF	1.093	0.870	0.927	1.111	1.190	0.935
rpsG	1.055	0.795	1.453	0.697	0.848	0.690
rpsL	1.124	0.982	1.410	0.485	0.845	0.710
rpsO	0.995	1.031	1.222	0.751	0.960	0.940
rpsP	1.130	1.183	1.071	0.617	1.095	1.130
rpsR	1.146	0.541	1.061	1.252	1.080	0.510
rpsS	1.107	1.087	0.981	0.826	1.140	1.115
rpsT	1.048	0.996	1.179	0.777	0.920	0.863
rpsU	0.561	0.817	1.220	1.402	0.460	0.670
guaB	0.883	0.835	1.115	1.166	0.795	0.750
pdhD	1.180	0.975	0.900	0.946	1.320	1.090
pdhA	1.346	0.838	0.895	0.921	1.534	0.943

pdhB	1.004	1.060	0.819	1.117	1.230	1.310
pdhC	1.221	1.057	1.003	0.719	1.253	1.107
ykyA	1.037	1.086	0.701	1.177	1.480	1.550
gltX	0.929	0.847	1.107	1.117	0.857	0.790
tyrS1	1.067	1.038	0.960	0.934	1.110	1.080
odhA	1.139	1.091	0.996	0.774	1.170	1.105
flgG	1.164	1.131	0.845	0.860	1.415	1.340
ylxF	1.037	1.037	0.746	1.179	1.390	1.390
lysA	1.014	0.872	0.948	1.166	1.070	0.920
menB	0.829	0.886	0.952	1.333	0.870	0.930
ctaD	0.820	1.209	0.828	1.143	0.990	1.460
oppD	1.104	1.279	1.000	0.616	1.100	1.280
oppF	1.278	1.269	0.969	0.484	1.320	1.310
oppC	0.998	1.189	0.868	0.946	1.150	1.370
oppA	1.028	1.129	0.942	0.902	1.094	1.203
recR	0.910	0.941	1.034	1.116	0.880	0.910
yaaK	0.909	1.017	0.988	1.086	0.920	1.030
prsA	1.036	1.008	0.997	0.959	1.050	1.016
yqxJ	0.903	1.032	1.290	0.774	0.700	0.800
mnmG	0.916	1.107	0.907	1.070	1.010	1.220
pyrE	1.623	0.827	1.047	0.503	1.550	0.790
pyrAA	1.374	0.871	1.018	0.737	1.352	0.892
pyrAB	1.580	0.698	1.156	0.566	1.420	0.620
spo0J	1.111	0.880	0.855	1.154	1.300	1.030
ansB	1.152	1.136	0.778	0.934	1.480	1.460
katA	0.908	0.852	1.270	0.970	0.715	0.670
rplU	1.227	1.047	0.994	0.732	1.247	1.053
srfAA	1.419	1.231	0.641	0.710	2.254	1.948
tpiA	0.869	1.006	0.933	1.191	0.936	1.080
spoVG	1.147	1.176	0.980	0.696	1.170	1.200
ftsA	0.884	0.867	0.842	1.406	1.050	1.030
secA	1.034	1.036	1.015	0.915	1.048	1.040
yvyD	0.825	1.031	0.897	1.247	0.920	1.150
groL	1.051	0.920	0.903	1.126	1.177	1.020
groS	0.790	0.963	0.868	1.380	0.910	1.110
rph	0.991	1.015	1.166	0.828	0.850	0.870
cheA	1.241	1.091	0.752	0.916	1.662	1.456
purA	1.440	0.892	0.905	0.764	1.590	0.987
guaA	0.844	0.902	0.942	1.312	0.900	0.960
ndk	0.826	1.224	0.978	0.972	0.857	1.253
aroC	0.882	0.923	1.026	1.169	0.860	0.900
hepT	1.035	0.965	1.005	0.995	1.030	0.960
cspB	0.791	1.182	1.084	0.943	0.730	1.090
hemE	0.815	0.993	1.481	0.711	0.550	0.670
hemH	0.663	1.020	1.055	1.263	0.630	0.970

hemY	1.241	0.738	0.868	1.154	1.430	0.850
degV	0.880	0.890	1.000	1.230	0.880	0.890
yvyE	0.824	1.186	0.980	1.010	0.840	1.210
nusA	0.889	0.919	1.010	1.182	0.880	0.910
rbfA	0.892	0.908	0.818	1.382	1.090	1.110
tuf	0.978	0.937	1.063	1.022	0.920	0.885
qoxB	1.191	0.933	0.923	0.952	1.290	1.010
qoxA	0.977	0.899	0.930	1.195	1.055	0.990
ppiB	0.807	1.023	0.971	1.199	0.845	1.055
rluB	1.238	1.070	0.945	0.747	1.323	1.140
rluB	1.217	1.002	0.973	0.808	1.250	1.030
resB	0.911	1.108	0.939	1.042	0.970	1.180
leuS	1.073	0.934	1.002	0.991	1.073	0.943
ffh	1.011	0.950	0.980	1.058	1.033	0.970
ilvC	1.274	1.016	0.891	0.820	1.430	1.140
rplI	0.798	0.909	1.134	1.160	0.700	0.795
ssbA	1.239	1.109	1.180	0.472	1.050	0.940
serS	1.016	1.048	1.008	0.927	1.015	1.038
metG	1.110	1.040	0.921	0.929	1.205	1.130
mfd	1.007	1.049	1.039	0.905	0.970	1.010
ftsH	1.082	0.765	1.128	1.025	1.073	0.703
lysS	1.121	0.918	1.077	0.884	1.047	0.897
walR	0.800	0.845	0.899	1.456	0.890	0.940
ppaC	0.865	1.107	0.993	1.035	0.875	1.120
yybJ	1.265	1.201	1.063	0.472	1.255	1.175
ychF	1.153	0.984	1.058	0.804	1.090	0.930
yyaE	0.886	0.965	0.995	1.154	0.890	0.970
yyaB	0.835	1.059	1.246	0.860	0.670	0.850
noc	0.992	1.013	1.034	0.961	0.960	0.980
pdxS	0.985	1.062	1.101	0.851	0.920	0.995
dck	1.008	0.977	1.028	0.987	0.980	0.950
yaaN	1.070	0.723	0.939	1.268	1.140	0.770
yaaT	1.067	1.026	1.036	0.870	1.030	0.990
rnmV	1.071	1.034	0.915	0.979	1.170	1.130
hslO	1.012	1.171	1.220	0.598	0.830	0.960
clpC	0.898	0.818	0.948	1.335	0.958	0.870
fhuD	1.228	1.046	0.830	0.896	1.480	1.260
rpmB	1.148	0.986	1.159	0.707	0.990	0.850
atpA	1.085	1.009	1.008	0.897	1.083	1.007
atpD	1.039	0.791	0.988	1.183	1.055	0.795
atpG	1.160	1.028	1.105	0.707	1.055	0.930
atpF	1.007	0.947	0.922	1.124	1.100	1.047
eno	1.057	0.937	0.882	1.124	1.215	1.070
rpoB	1.057	0.814	1.089	1.040	1.015	0.800
rpoC	1.051	1.042	1.033	0.874	1.065	1.068

ackA	1.084	1.087	1.240	0.589	0.870	0.875
cysK	0.942	0.942	0.897	1.220	1.050	1.050
bfmBAB	1.051	1.149	0.906	0.894	1.170	1.280
bfmBB	1.185	1.067	0.840	0.908	1.410	1.270
lonl	1.010	0.882	0.908	1.200	1.113	0.970
glpT	1.297	1.155	0.882	0.665	1.470	1.310
rocD	0.867	1.337	1.023	0.773	0.860	1.330
cmk	0.990	0.874	1.029	1.107	0.963	0.847
ypfD	0.924	0.839	1.003	1.234	0.918	0.842
clpQ	1.027	0.763	0.978	1.232	1.050	0.780
citZ	1.038	1.089	0.845	1.028	1.245	1.315
deoC	0.758	0.998	0.902	1.342	0.840	1.105
icd	1.028	1.169	1.062	0.741	0.980	1.120
rocF	0.788	1.371	1.023	0.818	0.770	1.340
pdp	0.902	1.032	1.030	1.036	0.885	1.010
glyA	1.160	1.037	0.901	0.903	1.290	1.155
upp	0.724	0.733	1.383	1.160	0.530	0.535
mcpA	1.249	1.149	0.664	0.938	1.897	1.770
mcpB	1.135	1.005	0.821	1.038	1.395	1.230
tcyC	0.932	0.951	0.971	1.146	0.960	0.980
galK	0.829	1.050	1.105	1.017	0.750	0.950
galT	0.870	1.121	0.966	1.043	0.900	1.160
pdxK	0.894	0.983	0.988	1.136	0.905	0.995
rocA	0.738	1.213	1.095	0.954	0.695	1.115
bacF	0.938	0.912	1.303	0.847	0.720	0.700
pta	0.750	0.749	1.520	0.980	0.547	0.517
abh	0.934	0.954	1.015	1.096	0.920	0.940
pyrR	1.220	0.987	1.038	0.755	1.175	0.950
pyrP	1.682	0.812	0.978	0.528	1.720	0.830
asnS	1.126	0.914	0.887	1.073	1.270	1.030
gpmI	0.834	1.127	1.127	0.913	0.740	1.000
clpY	1.069	0.792	1.028	1.111	1.040	0.770
treA	0.369	1.150	0.621	1.860	0.595	1.870
cotG	0.457	0.498	0.513	2.531	0.890	0.970
cheW	1.003	1.096	0.664	1.236	1.510	1.650
topA	1.059	0.968	0.905	1.068	1.170	1.070
aroA	0.736	0.737	1.377	1.150	0.535	0.535
ytxJ	0.558	0.700	0.946	1.797	0.590	0.740
cheC	1.023	1.038	0.752	1.188	1.360	1.380
feuA	0.611	0.604	1.511	1.274	0.413	0.420
murC	1.124	0.639	1.102	1.135	1.020	0.580
ytxH	0.661	0.862	0.890	1.587	0.740	0.977
pgk	0.817	0.921	0.950	1.312	0.871	0.973
cgeA	0.339	0.415	0.424	2.822	0.800	0.980
narG	0.948	1.211	0.751	1.090	1.345	1.700

teyA	0.960	0.887	1.138	1.015	0.845	0.780
yxiB	0.953	0.932	1.096	1.019	0.870	0.850
yxiE	0.864	1.015	1.005	1.116	0.860	1.010
yckB	0.787	0.758	1.147	1.308	0.690	0.660
hxlA	0.897	1.118	1.195	0.791	0.755	0.930
iolC	0.758	1.044	0.842	1.356	0.900	1.240
nasD	1.116	1.021	0.904	0.960	1.262	1.168
nasE	0.746	1.032	0.904	1.318	0.830	1.160
rplB	1.242	0.951	1.084	0.723	1.188	0.906
rplC	1.083	0.857	1.118	0.942	1.131	0.876
rplD	1.178	0.720	1.057	1.045	1.130	0.685
rplJ	1.346	0.988	0.961	0.705	1.410	1.030
rplW	1.041	0.871	1.596	0.492	0.835	0.680
pbpC	1.166	1.095	0.879	0.860	1.336	1.254
ahpF	0.890	0.913	1.065	1.132	0.835	0.855
mgsA	0.905	1.012	1.190	0.893	0.760	0.850
tkt	0.907	1.028	0.992	1.073	0.920	1.044
dhbC	0.990	1.068	0.971	0.971	1.020	1.100
dhbF	0.741	1.005	0.976	1.278	0.760	1.030
prfA	1.102	1.020	1.020	0.857	1.080	1.000
yqaP	1.161	1.224	0.965	0.650	1.203	1.270
htpG	1.166	1.148	0.791	0.895	1.480	1.450
licH	0.671	1.116	0.907	1.306	0.740	1.230
iolS	0.653	0.920	1.105	1.322	0.606	0.844
iolR	0.859	1.252	1.061	0.828	0.810	1.180
phoH	1.053	1.053	1.170	0.725	0.900	0.900
ybeY	0.821	0.986	1.016	1.178	0.823	0.977
drm	0.766	0.929	0.930	1.375	0.823	1.000
rplF	1.172	0.985	0.849	0.994	1.380	1.160
rplR	1.145	1.137	0.830	0.888	1.380	1.370
argS	0.916	1.024	1.006	1.054	0.910	1.020
ggaB	1.215	1.038	0.844	0.903	1.440	1.230
opuAC	0.828	0.832	1.066	1.274	0.775	0.780
efp	0.747	0.940	0.939	1.374	0.795	1.000
accC1	0.983	1.128	0.964	0.925	1.020	1.170
mdh	1.119	1.181	1.100	0.599	1.020	1.077
ykkA	0.920	1.055	0.844	1.181	1.090	1.250
nrdE	1.044	0.721	1.044	1.191	1.000	0.690
ypsC	1.197	1.254	0.877	0.671	1.370	1.430
ypwA	0.841	0.618	1.158	1.383	0.725	0.540
pnp	1.009	0.866	1.100	1.025	0.967	0.858
salA	1.188	0.926	0.873	1.013	1.360	1.060
clpX	1.174	0.952	1.080	0.795	1.106	0.886
cspD	0.677	1.258	1.613	0.452	0.420	0.780
ilvD	1.156	1.226	1.005	0.613	1.150	1.220

fabG	1.157	1.232	0.835	0.777	1.437	1.527
ftsY	1.130	1.030	1.008	0.831	1.130	1.038
panB	0.988	1.048	1.216	0.748	0.855	0.955
panC	0.878	1.062	0.924	1.136	0.950	1.150
aspB	1.013	1.046	0.944	0.996	1.088	1.123
ypoP	0.962	0.822	1.081	1.135	0.890	0.760
gcvT	0.790	1.104	0.954	1.152	0.830	1.160
glyQ	1.062	1.174	0.798	0.966	1.330	1.470
glyS	0.908	0.955	0.946	1.192	0.960	1.010
folD	1.147	0.846	1.064	0.943	1.087	0.800
ypiB	0.932	1.132	0.913	1.023	1.020	1.240
asnB	0.998	0.977	0.893	1.132	1.122	1.094
yrkN	0.738	0.816	0.971	1.476	0.760	0.840
yqeH	0.879	0.944	1.072	1.105	0.820	0.880
rsfS	0.944	0.903	1.026	1.128	0.920	0.880
yqeM	0.813	0.907	0.935	1.346	0.870	0.970
yqfL	1.111	0.953	1.046	0.890	1.065	0.905
trmK	0.812	0.835	1.176	1.176	0.690	0.710
yqfO	0.995	0.873	0.999	1.133	0.995	0.875
ispH	0.937	1.007	0.868	1.189	1.080	1.160
ispG	0.896	0.860	1.028	1.217	0.877	0.837
yqgA	0.796	0.946	1.075	1.183	0.740	0.880
glcK	0.798	0.883	0.849	1.469	0.940	1.040
yqhL	1.192	1.121	1.180	0.507	1.010	0.950
yqhY	0.892	1.048	0.866	1.195	1.030	1.210
yqiG	0.845	0.997	1.173	0.985	0.720	0.850
yqiS	0.970	1.046	0.844	1.139	1.150	1.240
yqiT	1.091	1.152	1.010	0.747	1.080	1.140
bfmBC	1.189	1.014	0.922	0.876	1.290	1.100
yqiW	0.832	0.862	1.003	1.303	0.830	0.860
yqjE	0.932	1.112	1.005	0.950	0.930	1.107
zwf	1.045	1.042	0.986	0.928	1.070	1.065
yqjL	1.043	0.913	0.621	1.422	1.680	1.470
nudF	0.764	0.913	1.090	1.233	0.703	0.840
mleN	1.063	1.121	0.837	0.979	1.270	1.340
mcpC	1.179	1.283	0.797	0.741	1.480	1.610
yhcH	1.075	0.925	1.156	0.844	0.930	0.800
yhcX	0.903	0.768	0.844	1.485	1.070	0.910
fabI	1.009	1.120	1.231	0.640	0.820	0.910
galE	0.942	1.213	1.290	0.555	0.730	0.940
infC	1.081	0.889	1.102	0.927	1.060	0.840
rpmI	0.505	0.828	2.020	0.646	0.250	0.410
bdbA	1.017	1.105	0.978	0.900	1.040	1.130
yorD	0.784	1.386	1.136	0.693	0.690	1.220
sunT	1.090	1.180	1.000	0.730	1.090	1.180

murAA	1.154	1.029	0.830	0.988	1.390	1.240
rplM	1.091	0.853	1.155	0.900	0.970	0.763
ywhB	0.798	0.971	1.156	1.075	0.690	0.840
ywhD	0.833	0.975	1.096	1.096	0.760	0.890
speE	0.845	0.777	1.257	1.121	0.670	0.610
albE	0.777	1.128	1.064	1.032	0.730	1.060
albA	0.774	1.097	1.594	0.535	0.485	0.690
fruA	1.065	0.725	0.895	1.315	1.190	0.810
yuaB	0.809	0.916	1.527	0.748	0.530	0.600
fabD	1.057	1.031	0.839	1.073	1.260	1.230
divIVA	1.029	0.974	0.917	1.080	1.123	1.060
ydaG	0.308	0.500	0.581	2.610	0.530	0.860
ahpC	0.976	1.115	1.343	0.566	0.730	0.840
greA	0.815	1.080	0.996	1.109	0.823	1.097
tig	1.014	0.975	1.016	0.996	1.000	0.963
tsf	0.986	1.024	1.052	0.938	0.949	0.973
gndA	1.008	0.916	1.089	0.987	0.930	0.840
yjlD	1.032	0.989	1.053	0.926	0.980	0.940
tpx	0.631	1.199	1.262	0.909	0.500	0.950
pgi	0.936	1.037	1.026	1.001	0.915	1.008
serC	0.893	1.159	1.299	0.648	0.690	0.895
sucD	0.911	1.076	1.140	0.873	0.800	0.954
sufC	1.167	0.952	1.026	0.856	1.140	0.927
fusA	1.174	1.047	1.128	0.651	1.060	0.923
yugI	1.076	0.898	0.891	1.134	1.205	1.020
yceD	0.903	0.941	0.950	1.207	0.950	0.990
metE	0.829	0.996	0.960	1.216	0.875	1.045
dps	0.818	0.739	0.985	1.458	0.830	0.750
trxB	1.053	0.939	1.132	0.877	0.930	0.830
pyk	1.143	1.083	1.085	0.688	1.055	0.995
sucC	0.984	1.113	1.085	0.818	0.923	1.040
frr	0.751	0.945	1.150	1.154	0.655	0.823
yodC	0.737	1.115	1.023	1.125	0.720	1.090
yxkC	1.372	1.032	0.867	0.729	1.603	1.193
cydA	0.892	1.248	1.274	0.586	0.700	0.980
yciC	0.710	1.135	0.887	1.268	0.800	1.280
yclQ	1.235	1.100	0.920	0.745	1.345	1.195
padR	1.005	0.934	1.015	1.046	0.990	0.920
fmt	0.965	0.928	0.928	1.179	1.040	1.000
ysaA	1.000	0.813	1.042	1.146	0.960	0.780
yscB	1.138	1.146	0.825	0.891	1.380	1.390
ysdB	1.145	0.855	0.806	1.194	1.420	1.060
ysdC	0.657	0.676	0.966	1.700	0.680	0.700
glcF	0.818	1.035	1.035	1.112	0.830	1.055
polX	1.114	1.184	0.995	0.706	1.120	1.190

mutSB	1.155	0.966	1.050	0.829	1.100	0.920
fadB	0.781	1.613	0.630	0.976	1.240	2.560
etfB	0.790	1.820	0.632	0.758	1.250	2.880
etfA	0.703	1.859	0.521	0.917	1.350	3.570
ysnA	0.786	1.025	0.995	1.194	0.790	1.030
ysnF	0.736	0.665	0.708	1.890	1.040	0.940
yvhJ	0.909	1.008	1.108	0.975	0.820	0.910
topB	0.827	0.868	1.356	0.949	0.610	0.640
ydaO	1.193	0.772	1.170	0.865	1.020	0.660
ddl	0.828	0.990	1.067	1.115	0.785	0.920
cshA	0.985	0.769	0.769	1.477	1.280	1.000
ydbS	1.012	1.072	0.993	0.923	1.020	1.080
ydbT	1.107	1.067	0.998	0.828	1.110	1.070
ydbT	1.007	0.930	0.969	1.094	1.040	0.960
immR	0.829	0.990	0.952	1.229	0.870	1.040
yddR	0.749	0.923	1.026	1.303	0.730	0.900
ywqH	0.615	0.581	0.581	2.224	1.060	1.000
capA	1.214	1.126	0.971	0.689	1.250	1.160
minC	0.875	1.008	1.018	1.099	0.860	0.990
mreB	1.123	0.994	0.864	1.019	1.300	1.150
jag	0.934	0.976	0.857	1.233	1.090	1.140
rho	1.064	0.976	0.887	1.073	1.200	1.100
murD	1.016	1.111	1.058	0.815	0.960	1.050
murE	0.992	1.070	0.957	0.982	1.040	1.120
srfAB	1.387	1.148	0.719	0.746	2.102	1.703
dapA	1.072	1.246	1.246	0.436	0.860	1.000
asd	0.962	0.968	0.832	1.238	1.328	1.308
ymxG	0.833	0.902	1.083	1.181	0.770	0.830
gtaB	0.916	0.670	0.916	1.497	1.013	0.743
valS	0.983	1.022	0.938	1.056	1.050	1.090
yacO	1.010	1.115	1.042	0.833	0.970	1.070
nusG	1.040	0.907	0.989	1.063	1.055	0.915
rplA	1.280	1.070	0.860	0.789	1.658	1.348
nrgB	0.713	0.855	0.950	1.482	0.750	0.900
wapI	0.975	1.154	0.995	0.876	0.980	1.160
ftsL	0.862	0.952	0.907	1.279	0.950	1.050
ald	0.992	1.120	1.067	0.821	0.930	1.050
patB	0.850	1.074	1.180	0.897	0.720	0.910
srfAC	1.553	1.294	0.588	0.565	2.640	2.200
alsT	1.225	1.013	0.881	0.881	1.390	1.150
ileS	1.039	1.061	1.003	0.897	1.038	1.065
rnjA	1.328	0.773	1.115	0.784	1.350	0.925
ykrA	1.110	1.073	0.917	0.899	1.210	1.170
defB	0.825	1.022	0.897	1.256	0.920	1.140
suhB	0.816	0.957	1.008	1.219	0.810	0.950

yydD	1.111	0.889	0.966	1.034	1.150	0.920
yycR	0.927	0.908	0.917	1.248	1.010	0.990
parE	1.058	1.029	1.129	0.783	0.945	0.925
pyc	1.127	1.002	1.003	0.868	1.125	1.000
yugH	1.080	1.059	1.070	0.791	1.010	0.990
ytfP	1.062	1.006	1.130	0.802	0.940	0.890
hemZ	1.021	1.032	1.053	0.895	0.970	0.980
ylzA	1.125	1.074	1.023	0.777	1.100	1.050
ppaX	1.124	1.285	0.899	0.692	1.250	1.430
fliG	1.264	0.998	0.739	0.998	1.710	1.350
AVERAGE	0.975	0.999	0.996	1.031	1.016	1.047
SD	0.18200842	0.16751457	0.16859041	0.28168643	0.267043022	0.358182517

increase
decrease
unknown

pta/S3610-2 Ratio	*acuA*/S3610-2 Ratio
0.784	0.716
2.156	2.750
0.856	0.910
0.864	0.764
0.901	0.809
1.065	1.078
0.888	1.092
0.667	0.675
1.240	1.010
0.784	0.816
1.615	1.213
0.841	1.333
0.659	0.739
0.516	0.667
0.993	0.980
0.856	0.954
0.964	0.873
0.711	0.732
0.669	0.669
0.951	0.981
1.046	1.192
0.644	0.962
0.840	0.943
1.045	0.995
0.977	1.106
0.808	1.000
0.930	0.930
0.748	0.729
0.905	0.958
0.925	1.054
0.694	0.975
0.561	0.904
1.042	1.128
0.881	0.931
0.660	0.791
1.324	1.365
0.855	0.842
0.271	0.332
1.214	1.402
0.683	0.598
0.827	0.924

1.308	1.212
1.022	1.200
0.980	0.869
0.835	0.883
0.802	0.809
0.251	0.268
0.782	0.880
0.967	1.077
0.914	0.768
0.401	0.580
1.625	1.557
0.939	1.017
0.899	1.222
0.992	1.021
1.246	1.118
1.071	1.041
0.954	0.823
1.184	0.981
1.463	1.134
0.882	0.723
0.857	1.045
0.758	0.725
1.275	1.283
1.616	1.312
0.711	0.739
0.743	0.865
1.062	0.834
0.957	0.958
1.057	1.170
0.633	0.868
0.530	0.742
1.863	1.753
1.338	1.468
0.763	1.495
1.050	1.309
1.224	1.553
0.657	0.741
1.590	1.333
1.189	0.968
1.864	1.530
0.763	1.298
0.853	0.798
0.825	0.839
0.851	0.802
0.858	1.025

1.092	0.862
0.497	0.521
0.776	0.866
0.690	0.957
1.089	0.868
1.174	1.449
0.536	0.598
1.207	1.199
0.992	1.091
0.620	0.648
1.014	1.092
0.960	0.912
0.512	0.570
0.935	2.870
2.630	6.741
0.636	0.793
0.604	0.854
0.788	1.232
0.226	0.255
0.818	0.691
0.821	0.791
1.051	1.354
0.608	0.814
0.876	1.010
0.456	0.720
0.795	0.973
1.319	1.044
0.248	0.496
1.542	1.288
1.234	1.531
1.217	1.717
0.690	0.796
0.669	0.562
0.799	0.799
1.527	1.384
1.727	1.705
1.047	1.212
0.642	0.660
0.639	0.728
1.492	1.492
1.039	1.029
0.778	0.932
0.951	1.127
1.667	1.655
1.078	1.007

0.718	0.915
1.190	0.957
2.294	1.705
1.113	0.878
0.781	0.829
0.921	0.931
1.000	1.000
0.864	0.936
1.375	1.389
0.937	1.045
0.778	1.178
0.426	0.504
1.129	0.880
1.024	0.956
1.036	1.325
1.098	1.283
0.973	1.884
0.870	1.083
1.726	1.345
1.177	1.010
0.704	0.750
1.227	1.244
0.991	0.929
1.139	1.043
1.363	1.263
0.787	0.761
1.169	1.082
0.822	1.038
0.821	0.780
0.744	0.830
1.000	1.080
1.877	2.070
1.154	1.011
0.828	0.815
0.975	1.017
0.936	0.915
1.072	1.047
1.092	1.423
0.802	0.897
0.694	0.573
1.752	1.534
1.446	1.457
1.240	1.430
1.046	1.055
1.164	0.975

2.443	1.066
0.963	0.845
0.662	0.662
0.838	1.038
0.435	0.660
0.914	0.859
0.170	0.173
1.309	1.283
0.828	1.000
1.015	0.791
0.771	1.049
0.753	0.821
0.901	0.886
0.716	0.793
1.491	2.116
1.549	1.231
1.140	1.215
2.095	1.846
0.771	0.992
0.724	0.776
0.992	0.941
0.784	0.753
2.441	2.060
0.958	0.836
1.486	1.343
1.425	1.358
2.141	2.118
1.143	1.012
0.550	0.788
1.334	1.057
1.115	1.051
1.444	1.123
1.085	1.145
0.979	0.752
1.148	1.047
0.768	1.010
1.128	1.244
1.130	1.033
1.120	0.934
0.779	0.779
1.448	1.343
0.613	0.889
1.074	1.042
1.374	1.319
0.791	0.869

0.771	1.021
1.419	0.829
1.354	1.465
0.542	0.742
0.894	0.849
0.692	0.797
0.517	0.583
0.560	0.770
1.034	0.915
0.982	0.937
1.012	0.997
0.700	0.794
1.098	1.374
0.832	0.660
1.042	0.906
0.928	0.995
1.429	1.810
1.049	1.265
1.120	1.026
1.564	1.339
0.879	1.020
0.399	0.601
0.738	1.357
1.338	0.825
1.278	1.001
0.908	0.990
0.701	0.774
1.680	1.280
0.697	0.715
1.646	1.337
2.341	1.334
1.644	1.418
1.310	1.047
0.992	0.804
1.670	1.209
2.320	2.119
1.319	1.419
1.854	1.971
0.915	0.432
1.361	1.343
1.443	1.336
0.400	0.583
0.782	0.743
1.323	1.135
1.545	0.971

0.914	0.956
1.927	1.644
0.881	0.923
0.851	0.770
1.198	1.155
1.556	1.491
1.370	1.375
0.880	0.880
0.870	0.748
0.621	0.664
0.717	1.058
2.052	2.298
2.640	2.620
1.055	1.257
1.216	1.339
0.815	0.843
0.836	0.936
1.132	1.112
1.167	1.333
0.856	1.034
3.229	1.646
2.396	1.338
2.875	1.253
0.963	0.763
1.233	1.217
0.943	0.897
1.805	1.549
2.019	1.772
0.775	0.873
1.648	1.690
0.629	0.617
1.259	1.294
0.662	0.827
0.973	0.871
0.572	0.698
1.197	1.225
1.395	1.224
1.913	1.213
0.644	0.689
0.879	1.339
0.754	0.789
1.040	0.970
0.839	1.253
1.146	1.396
0.532	0.821

1.075	0.639
0.715	0.724
0.816	1.175
0.752	0.778
0.645	0.657
1.016	0.962
1.283	1.007
0.827	0.779
0.710	0.866
1.667	1.431
1.506	1.241
0.874	1.063
1.165	0.987
0.972	0.921
1.554	1.239
0.794	0.917
2.625	2.350
1.211	1.273
1.200	1.120
1.122	1.171
1.215	0.942
1.668	1.228
0.549	0.580
0.846	1.083
2.674	2.553
1.434	1.224
0.767	0.836
0.971	1.232
1.032	1.054
1.161	1.249
1.021	0.990
0.844	0.570
1.226	1.179
1.093	1.056
1.694	1.959
0.712	0.644
1.370	1.167
1.623	1.393
1.224	1.137
0.888	0.700
1.786	1.589
1.004	0.930
0.951	0.838
1.040	0.795
1.372	1.392

2.981	2.839
0.772	0.772
1.190	1.302
1.306	1.176
0.843	0.742
2.002	1.751
1.158	1.793
0.951	0.844
0.802	0.712
0.833	0.619
1.010	1.060
0.569	0.752
1.485	1.692
0.963	1.675
0.885	1.011
1.285	1.149
0.628	0.635
1.397	1.282
1.093	0.970
0.814	0.831
0.815	1.033
0.833	1.074
0.789	0.871
0.936	1.632
1.108	1.077
0.800	0.884
0.852	0.870
1.694	1.373
3.185	1.537
1.050	0.851
0.914	1.235
0.963	0.713
0.206	0.635
0.181	0.197
0.812	0.887
0.992	0.907
0.642	0.641
0.311	0.389
0.861	0.873
0.481	0.470
0.990	0.563
0.433	0.558
0.725	0.830
0.120	0.147
0.884	1.135

0.961	0.888
0.935	0.914
0.775	0.910
0.607	0.592
1.264	1.655
0.559	0.770
1.260	1.130
0.587	0.803
1.775	1.341
1.332	1.099
1.254	0.778
1.981	1.423
2.066	1.747
1.424	1.333
0.813	0.843
1.013	1.133
0.884	1.009
1.020	1.100
0.580	0.786
1.286	1.190
1.808	1.899
1.316	1.313
0.514	0.854
0.498	0.702
1.038	1.513
1.452	1.452
0.695	0.838
0.563	0.680
1.179	0.991
1.290	1.280
0.876	0.974
1.346	1.150
0.674	0.674
0.564	0.727
1.063	1.219
2.000	2.049
0.779	0.893
0.877	0.605
1.792	1.869
0.610	0.445
0.997	0.861
1.172	0.914
1.621	1.279
1.500	2.786
1.885	2.000

1.508	1.606
1.614	1.474
1.320	1.404
0.772	0.935
1.144	1.177
0.848	0.724
0.702	0.981
1.099	1.215
0.762	0.802
1.277	0.956
0.911	1.107
0.893	0.872
0.500	0.553
0.796	0.854
0.836	0.800
0.604	0.674
1.358	1.207
0.690	0.710
0.881	0.770
0.788	0.847
0.744	0.722
0.673	0.800
0.543	0.601
2.349	2.209
0.746	0.877
0.857	1.012
0.852	0.919
1.459	1.541
1.358	1.158
0.638	0.662
1.050	1.245
1.231	1.232
0.734	0.642
0.631	0.752
1.085	1.145
1.591	1.731
1.274	1.096
0.608	0.517
1.577	1.750
1.698	2.186
1.187	0.980
0.781	1.281
1.130	1.228
1.131	2.000
1.493	1.616

1.168	1.042
1.219	0.950
0.742	0.903
0.760	0.890
0.915	0.871
0.753	1.093
1.448	2.096
0.810	0.551
1.082	1.224
0.984	0.961
0.977	0.934
0.118	0.192
1.772	1.997
0.740	0.979
1.113	1.044
1.743	2.090
1.087	0.935
1.114	1.068
0.694	1.319
1.021	1.194
1.378	1.788
1.078	1.263
1.376	1.137
2.336	2.278
0.947	0.795
0.748	0.780
0.682	0.819
0.561	0.507
1.213	1.073
2.657	2.724
1.236	1.403
0.689	0.872
0.655	0.991
2.111	1.561
1.522	2.130
0.559	0.895
1.660	1.475
0.961	0.893
0.819	0.787
0.873	0.709
1.278	1.287
0.959	0.716
0.386	0.398
0.736	0.931
1.577	1.676

1.392	1.165
0.800	1.652
1.042	2.400
0.767	2.028
0.658	0.858
0.390	0.352
0.932	1.034
0.871	0.914
1.378	0.892
0.861	1.080
0.667	0.521
1.097	1.161
1.337	1.289
0.920	0.850
0.674	0.806
0.575	0.709
0.277	0.261
1.761	1.634
0.796	0.917
1.102	0.975
0.757	0.792
0.992	0.909
1.247	1.364
1.021	1.097
2.083	1.699
2.457	2.857
0.866	0.878
0.714	0.790
0.620	0.452
0.943	0.981
1.213	1.338
0.994	0.877
1.753	1.453
0.481	0.577
1.114	1.318
0.674	0.745
1.208	1.364
0.947	1.197
2.750	2.292
1.390	1.150
1.206	1.215
1.707	0.956
1.235	1.194
0.657	0.814
0.669	0.785

1.075	0.860
0.743	0.728
1.397	1.346
1.347	1.201
1.365	1.338
1.324	1.254
1.141	1.153
1.447	1.382
1.623	1.857
1.267	1.000
1.073	1.094
0.462287682	0.479221897

1	mber of green rows
2	umber of red rows
3	No distinct rows
	Total number rows
